# A Review on Electrochemical Sensors and Biosensors Used in Assessing Antioxidant Activity

**DOI:** 10.3390/antiox11030584

**Published:** 2022-03-18

**Authors:** Irina Georgiana Munteanu, Constantin Apetrei

**Affiliations:** Department of Chemistry, Physics and Environment, Faculty of Sciences and Environment, “Dunărea de Jos” University of Galaţi, 47 Domneasca Street, 800008 Galaţi, Romania; georgiana.munteanu@ugal.ro

**Keywords:** antioxidant activity, sensors, biosensors, enzymes, DNA

## Abstract

Currently, there is growing interest in screening and quantifying antioxidants from biological samples in the quest for natural and effective antioxidants to combat free radical-related pathological complications. Antioxidants play an important role in human health and provide a defense against many diseases. Due to the valuable dietary role of these compounds, the analysis and determination of their amount in food is of particular importance. In recent years, many attempts have been made to provide simple, fast, and economical analytical approaches for the on-site detection and determination of antioxidant activity in food antioxidants. In this regard, electrochemical sensors and biosensors are considered promising tools for antioxidant research due to their high sensitivity, fast response time, and ease of miniaturization; thus, they are used in a variety of fields, including food analysis, drug screening, and toxicity research. Herein, we review the recent advances in sensors and biosensors for the detection of antioxidants, underlying principles, and emphasizing advantages, along with limitations regarding the ability to discriminate between the specific antioxidant or quantifying total antioxidant content. In this work, both direct and indirect methods for antioxidants detecting with electrochemical sensors and biosensors are analyzed in detail. This review aims to prove how electrochemical sensors and biosensors represent reliable alternatives to conventional methods for antioxidant analysis.

## 1. Introduction

During the last decades, the use of antioxidants has increased considerably in food industry. The majority of biochemical reactions ensure that life is associated with the production of free radicals which, in turn, favor oxidative stress and contribute to body deterioration. The complex biochemical pathways in the human body are responsible for countering oxidative stress through ensuring an adequate level of balance between prooxidants (free radicals) and antioxidants. The epidemiological data have indicated a inversed correlation between the intake of fruits and vegetables, which are naturally rich in antioxidants, and the incidence of certain diseases (cardiovascular disorders, metabolic illnesses, and cancer) [1].

The recent progress in medicine and in nutrition change the traditional approach to medical care into personalized medicine, which prioritizes the prevention of diseases and raises health awareness, mainly through lifestyle changes and approaches based on diet and nutrition [2]. In this context, the antioxidants from plants, such as flavonoids, vitamins, hormones, phenolic acids, and esters, are considered bioactive dietary compounds which can reduce oxidative stress, and have been associated with multiple health benefits [3]. Antioxidants play an important role in maintaining an optimum equilibrium in the human body [4], and the analysis of these compounds or of the antioxidant activity of various foods and beverages has determined the full ascension of this research topic [5]. Antioxidant activity and antioxidant capacity are terms which are frequently used alternatively, but the fact that they have different meanings needs to be emphasized. Antioxidant activity refers to the speed constant of the reaction between a certain antioxidant and a specific oxidant, while antioxidant capacity is a measure of the quantity (expressed in moles) of a certain type of free radical measured by a sample. The measurements of antioxidant capacity determine the quantity of a heterogeneous mix of antioxidants which react together to produce the total or the net capacity to neutralize a particular sample [6].

Antioxidants are molecules capable of inhibiting the oxidation of other molecules. From a nutritional point of view, an antioxidant is defined as any compound which, when present in low concentrations as compared to those of an oxidable substrate, significantly delays or inhibits the oxidation of that substrate [7].

Antioxidants may be classified in various ways. Based on their activity, they are grouped into two categories: enzymatic (for example catalase (CAT), superoxide dismutase (SOD), glutathione peroxidase (GSHPx), glutathione reductase (GRD), etc.) and non-enzymatic (for example selenium, coenzyme Q10, vitamin C, vitamin E, etc.) [8]. Enzymatic antioxidants function through decomposition and scavenging free radicals, and non-enzymatic ones operate through interrupting the chain reaction of free radicals [9].

Depending on their solubility, antioxidants may be classified by their solubility in water and solubility in lipids [10]. The antioxidants which are soluble in water (vitamin C, for instance) are present in cellular fluids, such as cytosol or the cytoplasmatic matrix. The antioxidants which are soluble in lipids (vitamin E, carotenoids, and lipoic acid, for instance) are predominantly located in cellular membranes [11].

**Figure 1:**
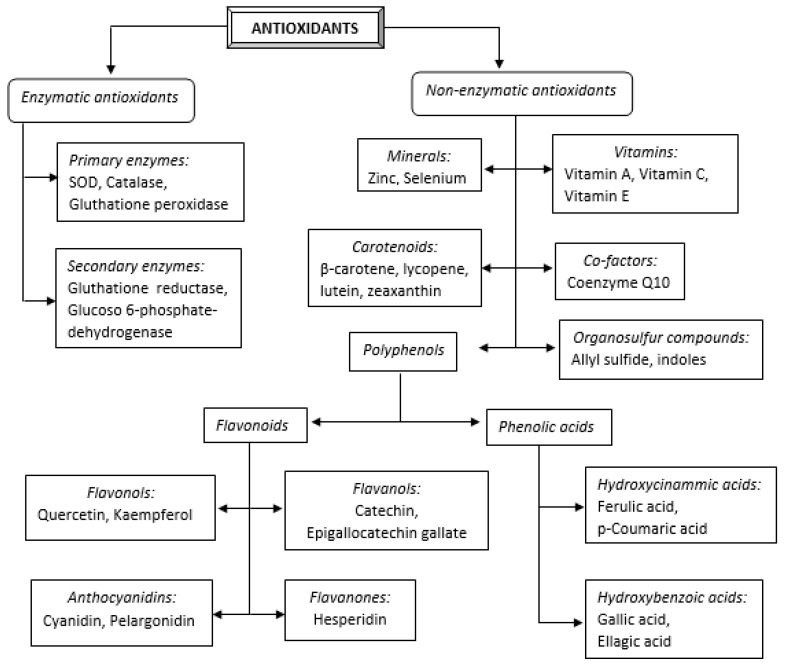
Classification of antioxidants.

The classification of antioxidants, together with the most representative examples of compounds in each class, is presented in Figure 1.

Antioxidant molecules can deactivate radicals through two major mechanisms: hydrogen atom transfer (HAT) and single electron transfer (SET) [12], producing the same final results, regardless of the mechanism. These two mechanisms almost always appear together in all samples, and the balance is determined by the pH and the antioxidant structure. The methods based on HAT measure the classical capacity of an antioxidant to neutralize free radicals through hydrogen donation (AH = H donor), according to the following equation:

$$X^{•\;}+\mathrm{AH}\to \mathrm{XH}+{\;A}^{•}$$

The methods based on SET detect the capacity of a potential antioxidant to transfer an electron, resulting in AH^•+^, and also to reduce any compound, followed by deprotonation in solution to form the corresponding, very stable A^•^ radical, as shown in the following two equations:

$$X^{•\;}+\mathrm{AH}\;\to X^{-}+{\mathrm{AH}}^{•+}$$

$${\mathrm{AH}}^{•+}+H_{2}O\;↔{\;A}^{•}+{\;H}_{3}O^{+}\;$$

Traditionally, the antioxidant activity can be measured using instrumental methods, such as gas chromatography (GC) [13,14], liquid chromatography (LC) [15,16,17,18], and colorimetry [19,20,21]. Although GC and LC are efficient techniques for separating and identifying antioxidants in complex samples, they require time, are costly, and need specialized personnel to operate. Colorimetry is an analytical technique used more frequently because it is easier to carry out, the costs are lower, and the time required by the analysis is shorter. These tests include the oxygen radical absorption capacity (ORAC) test [22,23], the total radical trapping antioxidant parameter (TRAP) test [24], the ferric reducing antioxidant power (FRAP) test [25,26,27,28], the cupric reducing antioxidant capacity (CUPRAC) assay [29,30,31], the 2,2’-azino-bis(3-ethylbenzothiazoline-6-sulfonic acid (ABTS) radical cation-based assay [32,33,34], and the DPPH (2,2-diphenyl-1-picrylhydrazyl) test [35,36,37,38]. The total content of phenolic compounds can be measured through the Folin-Ciocalteu (FC) test, which also reflects the antioxidant activity of the samples [39,40,41,42].

Although simpler than GC and LC, the colorimetric methods require a larger quantity of samples and reagents, are time-consuming manual processes, and, therefore, are not suitable for the rapid screening of the antioxidant activity. For instance, in the case of the ABTS test, one of the most important drawbacks is the time of reaction between ABTS^•+^ and the antioxidants. Some antioxidants react completely and almost instantly, while others react slowly or through a mixture of rapid and slow reactions; thus, different measurements are obtained, depending on the determination moment [43]. Hence, the reaction time necessary for the ABTS test should be taken into consideration on determining the antioxidant activity [44]. In the case of the DPPH test, the DPPH radical tends to react with other radicals which are present in the testing samples [45] and also the DPPH absorbance tends to decrease at light exposure, which requires an analysis in the dark [46]. The CUPRAC method cannot measure the antioxidant [47], and requires a longer time for measurement [48] and, sometimes, incubation at 50 °C in a water bath for 20 min for colored compounds [49].

In resolving these disadvantages and limitations of the colorimetric methods, the electroanalytical techniques based on sensors and biosensors have attracted attention, proving to be more rapid (real-time analysis), more sensitive, and more specific in relation to the compounds analyzed [50]. Sensors and biosensors have a series of advantages, including low cost, flexibility, portability, ease of use, possibility of remote use, rapid analysis time, reproducibility, long-term stability, a minimum need for pre-treatment of the sample, and possibility of miniaturization [51,52,53,54].

Researchers have reported various studies on determining the antioxidant activity through electrochemical sensors and biosensors, including comprehensive reviews [55,56,57,58,59,60,61]. This study presents recently published research on sensors and biosensors in view of recognizing and quantifying antioxidants in foods. It also brings together issues related to the advantages and disadvantages of these devices used for assessing antioxidant activity, as well as methods of correlation between (bio)sensor responses and the antioxidant character of compounds.

The review is organized in three sections. In the first part, we discuss the electrochemical sensors used in determining antioxidant activity and, in the second part, we discuss electrochemical biosensors for antioxidant activity determination, including enzymatic biosensors and also DNA-based biosensors. The third section describes the methods of correlation between (bio)sensors responses and the antioxidant character of the compounds.

## 2. Electrochemical Sensors for Determining Antioxidant Activity

Developing new sensors for the food sector is one of the essential areas in nanobiology and nanomaterials science. Special attention has been paid to the methods with high sensitivity, rapidity, low sample quantity required, as well as simple and economic instruments, in view of rationalizing the use of research resources [62]. Electrochemical sensors for the determination of antioxidants were developed using various types of electrodes, transducers, and receptors. In some cases, nanomaterials were integrated so as to obtain improved performances, thus increasing their sensitivity, stability, and selectivity [63]. The emergence and application of nanomaterials as an integral part of sensors had a visible impact on research. Nanomaterials are characterized by certain special thermic, mechanic, optic, electric, and magnetic properties, which depend on size and can be calibrated by the simple adjustment of shape, size, and degree of agglomeration [64]. These properties themselves and the effect of size are essential, which is manifested by the increase in electrochemical activity compared to that of the corresponding raw material [65].

In recent years, various analytical methods based on nanomaterials (including gold [66,67,68], silver [69,70,71,72], cerium oxide (CeO_2_) [73,74], nanoparticles, as well as the combination of CuO and ZnO, which can improve catalytic performances [75]) have been proposed to determine the antioxidant activity. The special properties of nanoparticles offer good perspectives for creating new efficient catalysts, sensor type devices, and medical systems, which have an increased biological activity and targeted delivery. When new functional materials based on nanostructures are developed, the results of the experimental studies on the effects of size and the related theoretical concepts proposed help to anticipate the properties of the newly created material. Knowing the kinetic and thermodynamic properties of nanoparticles themselves, and the processes which take place on their surface, may lead to the selection of better conditions for more efficient and more stable properties of electrochemical sensors [76].

The specialized literature presents the benefits of electrochemical methods in terms of sensitivity and resolution for determining the antioxidant activity with the application of nanostructured transducers [77,78,79,80]. Modifying the surface of the electrode with nanoparticles can reduce the oxidation overpotential of the antioxidant and can increase the peaks of their oxidation currents, leading to a significantly improved sensitivity and selectivity of the determination [81].

Electrochemical methods were used, with consideration of their advantages in relation to the possibility of rapidly proving the antioxidant activity of numerous organic compounds. The oxidation potentials, measured through cyclic voltammetry (CV), were used to compare the antioxidant power of various compounds, such as phenols, flavonoids, cinnamic acids, and tannins, frequently using a glassy carbon electrode (GCE) [82].

In this regard, the electrochemical behavior and the antioxidant efficacy of ascorbic acid, caffeic acid, quercetin, catechin, hesperidin, as well as binary equimolar mixtures, were evaluated using CV, in view of determining a prooxidant potential or a synergic behavior of antioxidant mixtures. An important synergic oxidation was noticed between quercetin and catechin. From among all the compounds and mixtures tested, the caffeic acid–ascorbic acid mixture showed the highest antioxidant tendency [83].

To determine the antioxidant activity of oenological tannins, Ricci et al., used two methods, namely the DPPH spectrophotometric method and the electrochemical method based on CV, and the obtained results obtained were compared thereafter. The CV measurements proved to be well correlated with the DPPH values due to the similarity of the chemical mechanisms which form the basis of both methods involving phenolic compounds as reducing agents. Moreover, the considerations on the extract composition can be derived from the voltametric profiles [84].

Photinon et al., reported the determination of polyphenols and their antioxidant activity in white wine, using an electrochemical sensor with a thick film, with an iridium–carbon working electrode. Caffeic acid was used as a model compound, since it has the capacity to produce the highest oxidation current. The correlation coefficient of 0.9975 was obtained between the sensor’s response and the caffeic acid content. The total content of phenolic compounds and the capacity to scavenge the DPPH radical were also correlated to the caffeic acid concentration, with a correlation coefficient of 0.9823 and 0.9958, respectively. The sensor prototype proved to be a simple, efficient, and cost-effective device to evaluate the antioxidant activity of polyphenolic compounds [85].

Flavonoids have also been studied for their antioxidant activity, both through classical methods and through electrochemical methods. Thus, in a study carried out by Firuzi et al., the antioxidant properties of various flavonoids in various subclasses (flavones, i.e., apigenin, baicalein, and chrysin; flavonols, i.e., fisetin, galangin, kaempferol, myricetin, quercetin, and rutin; flavanones, i.e., hesperetin, naringenin, and taxifolin; flavanols, i.e., catechin; and isoflavones, i.e., daidzein and genistein) were determined using an FRAP assay and their oxidation potential was determined through CV. A good correlation was noticed between the FRAP test and the electrochemical results. Most of the flavonoids tested in this study proved to be more active compared to known antioxidants, such as resveratrol, Trolox, and uric acid [86].

G.K. Ziyatdinova et al., carried out a study which aimed to evaluate the antioxidant properties of spices through CV. The individual antioxidants of spices (gallic acid, rosmarinic acid, capsaicin, thymol, and eugenol) are irreversibly oxidized between 0.88 and 1.25 V on the surface of a GCE in LiClO_4_ 0.1 M solution in ethanol. The detection limits and the quantification limits varied between 0.57 and 12 and between 1.8 and 40 µM, respectively. On the cyclic voltammograms of the spice methanolic extracts, distinct oxidation stages and peaks were noticed, and the potentials and areas of these peaks depended on the type of spice. The total surface of the oxidation stages was selected as a parameter which characterizes the antioxidant properties. The antioxidant activity of spices was expressed as a weight of gallic acid, in milligrams, corresponding to one gram of a dry spice. A good correlation was noticed between the antioxidant activity obtained through voltammetry and the FRAP test, the antiradical activity, and the total content of phenolic compounds. All the while, correlation coefficients varied between 0.8886 and 0.9615 [87].

Another study demonstrated the possibility of using square-wave voltammetry (SWV) and other electrochemical methods with screen-printed carbon electrodes for the quantification and evaluation of antioxidant activity, and of the quantity of specific antioxidants, mainly polyphenols, in certain fruit juices. Freshly squeezed cranberry and strawberry juices, from various types and maturation stages, were chosen depending on the known differences in their antioxidant profile. As a result of the increase in the potential applied (0–1.0 V vs. Ag/AgCl), the electroactive compounds present in the juices were oxidated, leading to a voltammetric profile which is characteristic for each of the samples analyzed. In general, the cranberry juice had higher oxidation peaks at lower potentials (<400 mV), indicating antioxidant activity. The relation between the cumulative responses of the sensor led to different applied potentials, and the total or individual antioxidants was evaluated, as determined through conventional spectrophotometric methods (FRAP, Folin-Ciocalteu) and HPLC, respectively, in the context of developing a rapid sensor to determine antioxidants [88].

Petković et al., used the differential pulse voltammetry (DPV) method to determine the gallic acid, using an electrochemical sensor based on immobilizing the binuclear copper (II) octa azamacrocyclic [Cu_2_tpmc](ClO_4_)_4_ complex in a PVC matrix and coated on graphite (CGE) or a carbon fiber tube (CGCE). The method proposed is based on the antioxidation process of gallic acid at the [Cu_2_tpmcGA]^3+^ complex on the surface of the electrode. The voltammograms, used as a supporting electrolyte, recorded a HNO_3_ solution of pH = 2.0, measured in the concentration range of 2.5 × 10^−7^ and 1.0 × 10^−4^ M gallic acid, which indicated two linear calibration curves (for the higher concentration range and the lower gallic acid one). The detection limit for CGE was 1.48 × 10^−7^ M and 4.6 × 10^−6^ M, respectively, for CGCE. The practical utility of CGE was demonstrated through estimating the antioxidant activity, expressed in gallic acid equivalents, in white, rosé, and red wine samples, using an extremely simple procedure, without any sample pre-treatment [89].

Gualandi et al., developed a new chemically modified electrode (CME) used to determine the antioxidant activity of some compounds, usually considered antioxidants, and of some fruit juices. The aim of the study was also to determine a correlation between the data obtained by the new sensor and those resulting from applying the ORAC, DPPH, and ABTS methods to the same samples. The best correlation was obtained with the ORAC values [90].

A direct determination of gallic acid was achieved with a carbon paste electrode modified with carbon nanotubes using DPV. The values obtained for gallic acid were used to estimate the antioxidant properties of some wine samples, based on gallic acid oxidation. In optimized experimental conditions, the calibration curve for gallic acid was linear in the concentration range from 5.0 × 10^−7^ to 1.5 × 10^−5^ mol·L^−1^, with a detection limit of 3.0 × 10^−7^ mol·L^−1^. The modified electrode obtained was successfully used to determine the antioxidant activity for the red and white wine samples, without the interference of glucose or ascorbic acid, and the results obtained were better as compared to those obtained through the standard spectrophotometric method in terms of selectivity, sensitivity, and quantity of waste produced [91].

Through the exploitation of the catalytic properties of gold nanoparticles, a specific and sensitive electrochemical sensor was developed, and the working parameters were optimized for screening the relative antioxidant capacity (RAC) of hydrosoluble plant extracts. Electrochemical determination methods (CV and amperometry) were used to characterize various sensors based on nanoparticles, determining the best performance, with a value of 98%, for lavender extracts.

The detection principle is based on the antioxidant neutralizing effect for the amperometric detection of H_2_O_2_, where a decrease in the electrochemical signal suggests an increase in the antioxidant activity. The results obtained were expressed in terms of ascorbic acid equivalents and Trolox equivalents in order to correlate these results with classical methods, such as chemiluminescence and UV–vis spectrophotometry. Furthermore, a correlation coefficient of 0.907 is obtained, suggesting a good correlation between electrochemical methods and spectrophotometric ones [92].

Recently, a method of obtaining a new sensor (G/PTH/N-GPH/GCE) through modifying a GCE with nitrogen-doped graphene (N-GPH), guanine (G), and polythionine (PTH) was described. This method had applicability in evaluating the antioxidant activity of natural and complex compounds, through the electrochemical method. The effects of pH, incubation time, guanine, and Fe^2+^ ion concentrations on the performances of the modified electrode were investigated and optimized. For this purpose, the effect of Fe^2+^ concentration on the oxidation peak current of the modified electrode was investigated following the dependency of the peak current on the Fe^2+^ concentration. It was found that the peak currents decreased with increasing of Fe^2+^ concentration to a maximum concentration of Fe^2+^, for which a small change in peak currents was observed. Hence, that value of the concentration was chosen as the optimum Fe^2+^ concentration for this study. Upon evaluating the antioxidant activity of ascorbic acid in optimum conditions, G/PTH/N-GPH/GCE showed good linearity, reproducibility, and stability. The antioxidant activity of three flavonoids and three plant extracts was determined using the new electrode obtained and the DPPH method. The highest value of the antioxidant activity was obtained for myricetin, through both methods. G/PTH/N-GPH/GCE had a series of advantages, including an easy fabrication procedure, a rapid detection time, and a low cost of determining the antioxidant activity of various sample types [93].

A novel, economic, and highly sensitive electrochemical sensor for the determination of trans-resveratrol (RES) was fabricated by electropolymerization of poly(L-lysine) films onto glassy carbon electrode (GCE) surfaces. Results showed that the detection range of the proposed sensor to RES was from 0.20 µM to 12.0 µM, with a minimum detectable concentration of 0.06 µM. The reported electrochemical sensor also exhibited high selectivity, good reproducibility, and long-term stability for RES detection [94].

Another study carried by Banica et al., was aimed to determine the total polyphenolic content and the antioxidant activity of commercial food supplements containing extracts of *Echinacea purpurea*, through DPV, using both an unmodified GCE and two GCE modified with carbon nanotubes (CNTs). A good correlation between the antioxidant activity and the total phenolic content was achieved, demonstrating the importance of polyphenolic compounds that contributed to the antioxidant activity of *Echinacea* extracts, but also to the total antioxidant effect [95].

The antioxidant activity, total phenolic compounds, and β-carotene content of orange-fleshed fruits and vegetables, including carrots, persimmons, and pumpkins, were evaluated by standard and electrochemical methods. The antioxidant activity was evaluated by the ABTS and DPPH measurements and the total phenolic compounds was determined spectrophotometrically. Electrochemical measurements were performed with differential pulse voltammetry at a sensor formed by attaching single-walled carbon nanotubes onto the glassy carbon electrode surface. Regression analysis was performed to correlate the results of the spectrophotometric assays with those obtained by electrochemical methods, with satisfactory results [96].

Platinum nanoparticles are also included in the category of nanomaterials, which were used to determine antioxidants in foods. In this regard, Romero et al., evaluated the antioxidant activity of tea extracts using DPPH, CUPRAC, and two electrochemical approaches, revealing that radicals were generated from hydrogen peroxide, using a mercury electrode and GCE protected with platinum nanoparticles and poly-neutral red (PNR-Pt). The LOD value obtained for this method was 17.2 µg·g^−1^. There were good correlations between the antioxidant capacities measured through the two electrochemical techniques, and between these techniques and CUPRAC, but the DPPH radical scavenging test measured the antioxidant activity differently than the rest of the methods. Therefore, the total content of antioxidants for an extract is not linked to its antioxidant activity since the distinct components of extracts may have very different antioxidant capacities [97].

Table 1 lists several examples of sensor-based electrochemical methods for the determination of antioxidants.

### Advantages and Disadvantages of Electrochemical Sensors

Developing new sensors with applicability in the food sector and/or the pharmaceutical sector is one of the essential areas for nanotechnology and materials science. Special attention has been given to simple, rapid methods with high sensitivity, though only a small quantity of samples was necessary for analysis, with the aim of rationalizing the use of research resources [62]. In this regard, nanomaterials were used to develop numerous sensors and detection methods. Nanomaterials are used as catalytic instruments to improve the performances of detection, highlighting their high sensitivity, selectivity, and stability [98]. By contrast with the conventional tests for the detection of antioxidant activity, electrochemical sensors have a series of advantages, such as portability, low cost, ease of use, rapid responses, high sensitivity, long-term stability, reproducibility, uncomplicated specialized equipment, and the expandability of pre-treating samples for analysis [99,100]. Although nanomaterial-based electrochemical sensors have improved the analysis sensitivity, they can respond to a wide range of compounds. Therefore, in most cases, they are unable to discriminate between responses of compounds with similar electroactive functional groups in their structures, especially in case of biological samples, which have very complex nature, due to their wide range of organic and inorganic compounds [101].

## 3. Electrochemical Biosensors for Determining Antioxidant Activity

The importance and necessity of using biosensors is constantly growing, since the integration of innovative materials can improve their performance, in terms of sensitivity and specificity. The sensitivity of a biosensor depends on the type of transducer (electrochemical or optical) and on the technique used to immobilize or functionalize various nanomaterials or polymers which amplify the output signal. Selectivity and specificity depend on the choice of the materials used and on the specific recognition elements, such as enzymes or DNA [102]. Biological recognition elements, such as enzymes, aptamers, DNA/RNA, and cells (bacteria, plant cells), are the key components of electrochemical biosensors [103].

Monitoring antioxidant activity through electrochemical biosensors, based on the redox principle, has many advantages compared to the conventional chemical methods and is commonly used for the initial screening of antioxidants. This technology does not require chemical reagents or sophisticated solvents, nor does it require special treatment of samples. It offers extended and reproducible information about electrodynamic processes and ensures a rapid achievement of determinations [104].

### 3.1. Enzymatic Biosensors

Enzyme-based electrochemical biosensors use enzymes as a biorecognition element and the sample analysis is based on the inhibition of enzymatic activity. After the enzyme is exposed to a certain inhibitor for a certain period of time, quantitative and qualitative analyses of the analytes are performed by determining the correlation between the enzyme inhibition rate and that of the inhibitor concentration [105].

**Figure 2:**
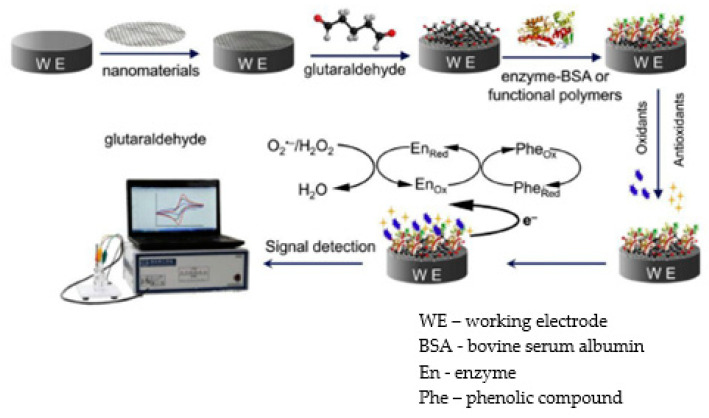
The schematic representation of developing a modified electrode based on enzymes. Published from [102] with the permission of the publisher.

The schematic representation of preparing a biosensor using enzymes and the detection mechanism is presented in Figure 2.

Enzyme-based biosensors have several advantages related to the nature of the enzyme. They are highly selective for a particular substrate and, for a large number of substrate molecules, reactions can be catalyzed by a single enzyme molecule, resulting in an amplification of the effect and an increase in sensitivity [106]. The enzymes commonly used in developing biosensors belong to the oxidoreductase, hydrolase, or lyase categories. At present, a variety of proteases are used to determine antioxidants and to evaluate their activity through biochemical oxidation, followed by electrochemical reduction [107]. Tyrosinase [108], laccase [109], peroxidase [110], and other proteases with simple or complex enzymatic bindings [111] are among them. The electric coupling of oxidoreductase and the electrochemical transducer have excellent characteristics, and monitoring is achieved through controlling the enzyme reaction in real time [106]. Specific enzymes can be used efficiently for the selective identification of important target compounds in food quality control. Laccase and tyrosinase are the two enzymes which are most frequently used to monitor antioxidants, especially phenolic compounds [112].

#### 3.1.1. Electrochemical Biosensors Based on Tyrosinase

**Figure 3:**
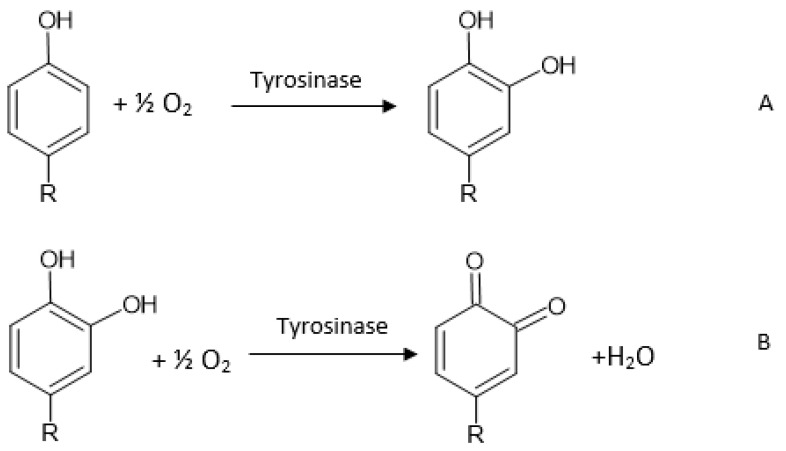
Tyrosinase reaction mechanism (**A** = cresolasic activity; **B** = catecholasic activity).

**Figure 4:**
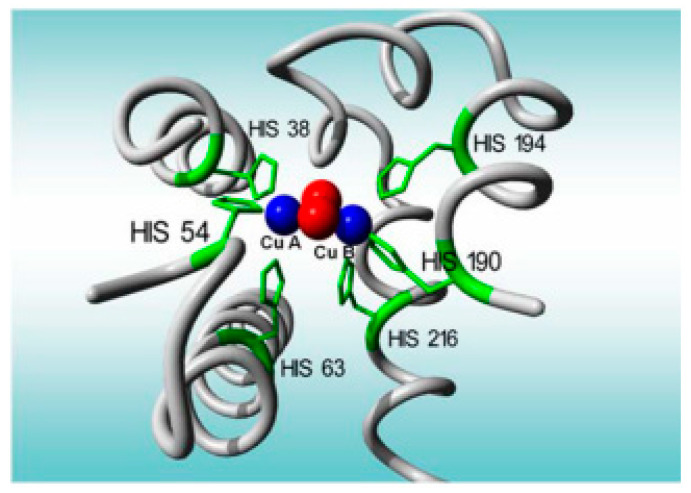
Active sites of the tyrosinase enzyme [73].

Tyrosinase is an enzyme which has two characteristic catalytic sites: one for phenol hydroxylation (cresolasic activity) and one for diphenol oxidation, up to quinone (catecholasic activity). Furthermore, both sites are active in the presence of molecular oxygen (Figure 3). Tyrosinase is a metalloenzyme which has two copper ions at the level of the active enzymatic site, each coordinated through three histidine residues in the enzymatic polypeptide chain (Figure 4) [113]. The oxidoreductase action of tyrosinase is ensured by the reversible transfer of electrons through copper ions (${\mathrm{Cu}}^{+}↔{\;\mathrm{Cu}}^{2+}$) [108]. Tyrosinase was immobilized on various electrodes, as well as in combination with a variety of nanomaterials, in order to develop electrochemical biosensors for the analysis of antioxidant activity. Various types of nanomaterials such as metallic nanoparticles and metal oxide nanoparticles (gold, silver, and platinum), as well as carbon structures (graphene and carbon nanotubes), were used to increase the electrochemical performances of the biosensors based on tyrosinase [114].

The tyrosinase enzyme is most frequently used for achieving biosensors to determine phenols in food samples.

Rodríguez-Sevilla et al., described a simple construction of a biosensor. They immobilized tyrosinase (Tyr) from mushrooms on screen-printed electrodes (SPEs) using three different techniques: capturing with polyvinyl acid (PVA) soluble in water and crosslinking with glutaraldehyde (GA), in the absence and in the presence of human serum albumin (HAS). The electrodes obtained were the following: SPE/Tyr/PVA, SPE/Tyr/GA, and SPE/Tyr/HSA/GA. All the configurations of biosensors in the presence of catechol were tested through amperometry and electrochemical impedance spectroscopy (EIS) techniques. It was noticed that the best performances were obtained for SPE/Tyr/GA, with a sensitivity of 26 ± 4 nA·µM^−1^. Finally, the biosensor was used to quantify the antioxidant activity, under the form of Trolox equivalents, in medicinal plant samples [115].

Another study described an amperometric biosensor for determining hydroquinone and other phenolic antioxidants, based on the carbon paste electrode on which tyrosinase is immobilized in a Nafion film. The tyrosinase in mushrooms was used to catalyze the oxidation of p-hydroquinone, whereby satisfactory values of LOD, i.e., 1.6 µM, were obtained. For the interference study, ascorbic acid was used as an interference because this compound can reduce quinone generated by enzymatic reaction in higher concentrations. It has been observed that increasing the ascorbic acid to hydroquinone ratio results in a linear decrease in the current response of up to 80%. Hence, it could be concluded that in samples analyzed with the tyrosinase-modified CPE, the ascorbic acid to hydroquinone molar concentration ratio should not exceed 1. For the experiments connected to substrate specificity, catechol, resorcinol, phenol, and Trolox were used, and the authors concluded that Tyr predominantly catalyzes the oxidation of polyphenols which have the hydroxyl cluster in ortho position. The compounds with the cluster in a meta or para position require a longer oxidation time, thus allowing the use of the biosensor to determine the total antioxidant capacities in wine samples [116].

Sỳs et al., described the use of an amperometric biosensor based on a carbon paste electrode coated with a thin layer of carbon nanotubes and a Nafion film containing the tyrosinase enzyme to directly determine the antioxidant activity expressed in Trolox equivalents (TEAC) in selected samples of *Moravian* wines. The results obtained were compared to the DPPH spectrophotometric method. Although the two methods are based on different principles, their results were comparable, with a correlation coefficient of 0.9752 [117].

Moreover, TEAC was determined for various berries, using a carbon paste electrode with multi-walled carbon nanotubes, coated with a layer of Nafion and containing the tyrosinase enzyme. The electrochemical behavior of the biosensor and the influence of multi-walled carbon nanotubes were studied in relation to the sensitive amperometric detection of the total content of phenolic compounds in berries, expressed as Trolox equivalents. After the optimization of the electrolytic parameters, the biosensor was used to determine TEAC in blackberries, blueberries, raspberries, and strawberries by the method of multiple standard additions. It was noticed that the electrochemical TEAC assays corresponded well with results obtained by the DPPH assay.

**Figure 5:**
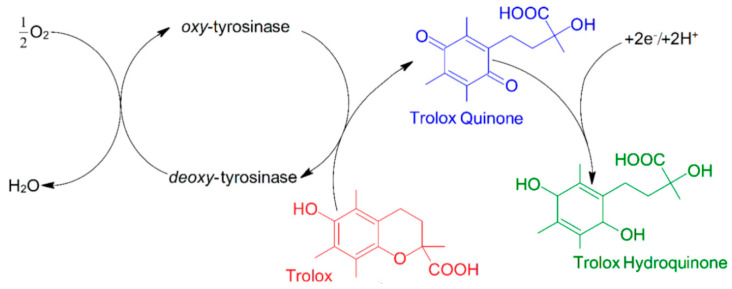
Principle of amperometric biosensor based on tyrosinase for TEAC evaluation [118].

Figure 5 shows the principle of the reactions involved in determining TEAC with the aid of the biosensor.

#### 3.1.2. Electrochemical Biosensors Based on Laccase

**Figure 6:**
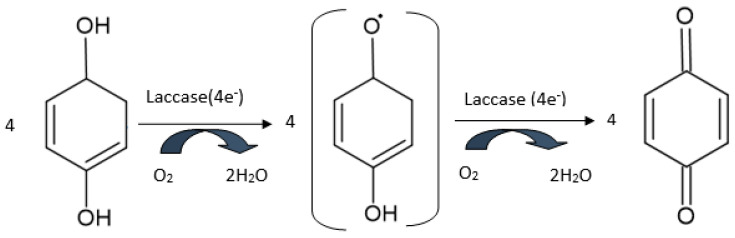
The oxidation reaction of phenolic compounds catalyzed by the laccase enzyme.

Laccase belongs to the multi-copper oxidase (MCO) family, i.e., a group which includes many enzymes with different specificities for different substrate and various biological functions. Lacasse is made up of four copper atoms (copper type 1; copper type 2; and two copper type 3 atoms), which form the active site of the enzyme [119]. This enzyme catalyzes the oxidation of phenols, diphenols, and other polyphenols at quinone derivatives, without requiring H_2_O_2_ as a co-substrate [120]. Figure 6 shows the oxidation mechanism of phenolic compounds by laccase. Similarly, with the biosensors based on tyrosinase, reducing the generated quinone enzymatic derivatives supplies the electrochemical signal for the obtained biosensors [109]. In fact, the antioxidant activity is measured using a standard compound-like caffeic acid, catechin, chlorogenic acid, or catechol, and the respective compound displays good electrochemical behavior on the surface of the electrode [121]. From among the redox enzymes, laccase has very good stability, which makes it ideal for antioxidant analysis.

In this regard, a study was carried out to obtain a modified carbon paste biosensor based on laccase by mixing a raw *Pycnoporus sanguineus* laccase extract with graphite and mineral oil. The biosensor was capable of detecting the total phenol compound content in red fruit extracts. Moreover, the study characterized the antioxidant profile of these red fruit extracts through CV, SWV, and DPV. The antioxidant potential, expressed through an electrochemical index (EI), was compared to the results obtained through the DPPH method. The results obtained demonstrated a good correlation between the total phenolic compound content and the antioxidant potential, and a significant similitude of the results obtained through the three methods, which both justify the electrochemical approaches as instruments for quality control and for the antioxidant characterization of natural products [107].

de Oliveira Neto et al., developed a modified carbon paste biosensor based on laccase to determine the total phenolic compound content and the antioxidant activity in honey [122]. The results obtained with the biosensor showed an acceptable association with the classical FRAP and DPPH determination methods. The test was rapid, with a detection time of less than 30 s, in accordance with the time necessary for the enzymatic oxidation of phenolic mixtures.

Like tyrosinase-based biosensors, nanomaterials can be integrated into laccase-based biosensors in order to improve their sensitivity. Thus, a study carried out recently described the obtaining of a simple and very sensitive electrochemical biosensor based on laccase immobilized on the surface of the screen-printed carbon electrode (SPCE) modified with graphene nanoplatelets (GNPI) and gold nanoparticles (AuNP). The biosensor obtained, LACC/AuNP/GNPl/SPCE, was used in the amperometric determination of hydroquinone and other phenolic compounds. GNPI, 2D carbon nanomaterials with better thermic and mechanic qualities, as well as superior electric characteristics by comparison with other carbon nanomaterials, act as “electronic wires”. These wires ensure a shorter movement of the electrons in the prosthetic groups located in the enzyme structure, in depth, and protect the protein from the adsorptive denaturation on the electrodes or from undesired inclinations of the molecules. This characteristic turns them into an ideal substrate for immobilizing redox enzymes and for fabricating electrochemical biosensors. AuNPs/GNPI accelerated the movement of the electrons between the electroactive site of the enzyme and the surface of the electrode, and facilitated the orientation of the molecules to determine the phenolic compounds. The proposed biosensor indicated a linear range for hydroquinone from 4 to 130 µM, with a detection limit of 1.5 µM. The biosensor had good repeatability, reproducibility, long-term stability, and increased selectivity as compared to hydroquinone, and was used to determine the antioxidant activity of wine and cranberry syrup. The results were in keeping with those obtained through the standard method for determining antioxidant activity, expressed in Trolox equivalents [123].

**Figure 7:**
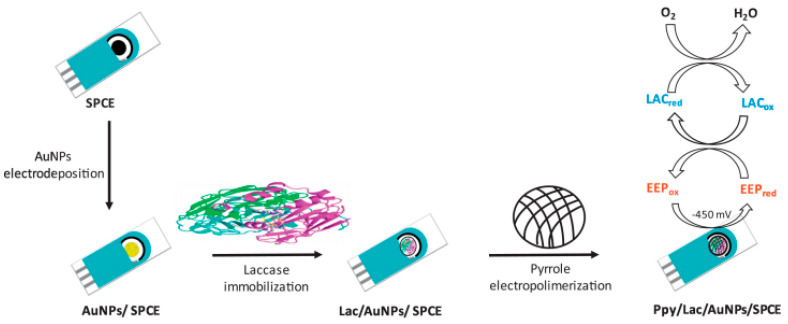
A schematic representation of SPCE modified with a Ppy/Lac/AuNPs nanocomposite film for the quantification of polyphenols in propolis samples. Published from [124] with the permission of the publisher.

Another study was aimed to develop an electrochemical biosensor for the determination of polyphenols in propolis samples. The construction of the biosensor is based on immobilizing a nanocomposite film of the laccase enzyme on AuNP, electrodeposited on the surface of a SPCE modified with polypyrrole (Ppy) through a process of in situ electro polymerization (Figure 7). The electrodepositing of gold nanoparticles facilitated an increase in the surface available for immobilizing laccase. The Ppy/Lac/AuNPs/SPCE nanocomposite film was characterized by electronic scanning microscopy and energy-dispersive X-ray spectroscopy, as well as through CV. In the presence of the propolis extract, which contains phenolic compounds, immobilized laccase oxidates the polyphenols and, later, these compounds are reduced on the surface of the electrode modified through amperometry at −450 mV. The linear range was between 1 and 250 µM, expressed as caffeic acid, and the detection limit was 0.83 µM. The time necessary for the analysis was 15 min, which was shorter compared to the time necessary for the spectrophotometric methods (85 min), especially for the Folin-Ciocalteu method. The biosensor developed had good selectivity, stability, and reproducibility which helped to detect polyphenols in the propolis samples [124].

García-Guzmán et al., described the achievements of a biosensor through the modification of the sonogel–carbon electrode with laccase extracted from *Trametes versicolor* mixed with glutaraldehyde and Nafion, and the obtained solution was added drop by drop on the surface of the electrode. The phenolic compounds in wines were analyzed, firstly to detect individual phenolic compounds (gallic acid, quercetin, rutin, and tannic acid, ferulic acid; (+) catechin; (−) epicatechin (ECAT); tyrosol; caffeic acid (CA); vanillic acid; syringic acid; p-coumaric acid; and 4-methylcatechol) and, secondly, to determine the total content of polyphenols. From the first test, the authors discovered that not all the polyphenols selected provided an amperometric response, but that good sensitivities were obtained for the majority of o-diphenols (LOD = 0.011 mg·L^−1^). For the second test, the increase in the signal as compared to the signal obtained for individual phenols was explained either through the synergic effect among polyphenols or through the individual contribution of several undetectable polyphenols. At the same time, this correlation study determined the antioxidant activity through the ABTS spectrophotometric method in order to clarify the influence of both polyphenols and sulfur dioxide on the stability of wines. Good correlations were found between the polyphenol index and the antioxidant activity, and poor correlations were found between the concentration of sulfur dioxide and the antioxidant activity [125].

In another study, multiple configurations of biosensors were tested and compared using both laccase from *Trametes versicolor*, and tyrosinase from mushrooms, immobilized on a glassy carbon electrode, using various agents, including bovine serum albumin (BSA) and GA as crosslinking agents, as well as chitosan and Nafion. The biosensor based on Lac had a better performance for the detection of catechol, and the configuration of the biosensor was optimized (the glassy carbon electrode was modified with a mixture of reduced graphene oxide and multi-walled carbon nanotubes (as a hybrid layer), followed by the laccase immobilization stage). To prolong the life of the biosensor, several protective biomembranes were tested, and a mixture of 20 mg·mL^−1^ BSA and 2.5% (*v*/*v*) GA, noted with BSA-GA1, proved to be optimal. Finally, the chronoamperometric response of the biosensor was recorded at 0 V, and the calibration curves were constructed through the graphic representation of the reduction current depending on the concentration of catechol. The performance of the GCE/hybrid/Lac/BSA-GA1 biosensor was tested for a variety of polyphenols (gallic acid, pyrogallol, 2,3-dihydroxibenzoic acid, dopamine, epicatechin, catechin, rutin, caffeic acid, and chlorogenic acid), manifesting good sensitivity in relation to the majority of the compounds. For the analysis of real samples, fruit juices were analyzed, comparing the results obtained with biosensors based on Lac and Tyr for the total content of polyphenols. Moreover, this study estimated the antioxidant activity using the standardized ABTS method to establish if it can be linked to the total content of polyphenols. From the data obtained using the two biosensors on real samples, it can be deduced that the antioxidant activity is mainly attributed to the total polyphenol content, as the same order of magnitude is obtained for all determinations [126].

#### 3.1.3. Electrochemical Biosensors Based on Laccase–Tyrosinase

Biosensors based on the co-immobilization of both laccase and tyrosinase were developed to extend the range of the detected polyphenolic compounds, due to the different catalytic activities of these enzymes. Nevertheless, there is a limited number of studies on this type of biosensors, mostly focused on determining the content of polyphenol compounds, later achieving a correlation between the polyphenols in the samples and the antioxidant activity of those samples, determined through classical methods.

The first biosensor based on laccase–tyrosinase was manufactured by ElKaoutit et al., and applied to monitor the total polyphenol index in several beer [127]. The biosensor was prepared through doping a sonogel–carbon electrode with a solution containing the enzymes, glutaric dialdehyde, and a Nafion proton-changing membrane. The analytical performances of this bienzymatic biosensor improved in comparison to those of the mono-enzymatic one, made by the same group. In both cases, the results obtained with the biosensors were correlated with those obtained through the Folin-Ciocalteu method.

In another study, an amperometric biosensor was developed through immobilizing the two enzymes, tyrosinase and laccase, on graphite screen-printed electrodes modified with ferrocene determine polyphenols in wine. By determining the best conditions and the adequate analysis of the samples, various immobilization procedures were performed, and the operating parameters of the sensor were optimized. Laccase has been co-immobilized with tyrosinase in a sol–gel matrix of diglyceryl silane (DGS) with the aim of widening the range of detected phenolic compounds, due to the different catalytic activity of the enzymes. The biosensor was tested on wine and wine samples of different varieties and from different regions. Spectrophotometric analyses of the samples, the Folin-Ciocalteu method (the official method for the analysis of polyphenols in wine), and measuring the absorbance of wines at 280 nm were all carried out in order to compare the results obtained with the biosensor with those obtained from reference methods. The biosensor provided good results when it was used to analyze wine, indicating good concordance with the spectrophotometric data obtained through the Folin-Ciocalteu method. For the purpose of interfering studies, sulphite and sulfur dioxide were used as interfering compounds. These compounds act as inhibitors of catalytic activity of both laccase and tyrosinase and, consequently, they seriously compromise the biosensor responses. Thus, supplementary studies are necessary to establish the best conditions for obtaining results which are uninfluenced by the presence of sulfur dioxide [128].

Diaconu et al., developed a bienzymatic biosensor to determine the total content of polyphenols in plants. An indium–tin oxide electrode was modified with multi-walled carbon nanotubes, and the laccase and tyrosinase enzymes were immobilized in a chitosan matrix. Soiling the surface of the biosensor was avoided by applying the Tween 20 surfactant. The data registered in a medium containing surfactant indicated a significant improvement in the operational stability and an extended linear range. The biosensor was used to evaluate the total content of phenols in *Salvia officinalis* extracts and in *Basilicum callus* cultures [129].

#### 3.1.4. Electrochemical Biosensors Based on Peroxidase

**Figure 8:**
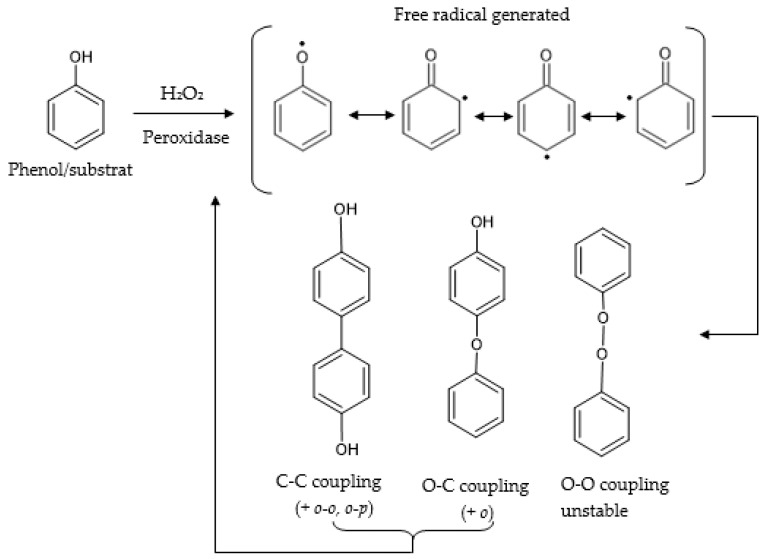
The mechanism of the oxidation reaction of phenolic compounds catalyzed by the peroxidase enzyme.

Peroxidases are enzymes which catalyze the oxidation–reduction reactions by the free radical mechanism (Figure 8) [130]. They convert substrates into oxidized or polymerized products. Horseradish peroxidase (HRP) is one of the most commonly used peroxidases in both biochemistry and biotechnology applications. However, there are only a few studies regarding electrochemical biosensors based on peroxidase in the determination of antioxidants.

Mello et al., presented a biosensor based on HRP, immobilized on a carbon paste electrode in a silicon–titanium matrix to determine phenolic compounds in teas and coffee [131]. The results obtained were compared with the traditional Folin-Ciocalteu method, and were well correlated with the latter. Furthermore, a good correlation was obtained between the total content of phenolic compounds determined with this biosensor and the total antioxidant activity, following the DPPH radical reduction method [132].

Similar to the previous study, Mello et al., evaluated the relationship between the total antioxidant activity and the phenolic compound content of Yerba Mate extracts, using the same HRP-based biosensor described and used in that study. The antioxidant activities of the extracts were investigated through the DPPH radical reduction method. The total antioxidant activity of the extracts was well correlated with the content of phenolic compounds, and the correlation coefficient was R > 0.9. The antioxidant activity, expressed in terms of the relative antioxidant activity of different origin Yerba Mate extracts, was determined in relation to the 10 mmol·L^−1^ Trolox solution. According to the results obtained, it was concluded that the simple use of the biosensor provides insight into the total antioxidant activity of various samples, which then presents a series of advantages, such as easy manipulation, a selective response, and a rapid evaluation of the antioxidant activity of plant extracts [133].

Table 2 includes several examples of electrochemical biosensors based on enzymes developed for the determination of antioxidants in food.

#### 3.1.5. Advantages and Disadvantages of Enzymatic Biosensors

Major progress regarding biosensors based on enzymes is linked to the immobilization and functionality of the biological material interface on the surface of the electrode [134]. Despite the good performances and the significance of this technology for applied and fundamental science, it should be noted that there are several important aspects which need to be taken into consideration before the commercial application of enzymatic biosensors to monitor active compounds and their antioxidant activity. One such aspect is related to the immobilization and the stability of the enzyme. Very efficient enzymatic immobilization and acceleration of the electron transfer rate are challenging tasks in developing biosensors. Along these lines, for an enzymatic immobilization with high efficiency and long-term stability, various nanomaterials or polymeric membranes, established and selected depending on the desired effects, can be integrated in biosensors [135].

Another important parameter is related to the biocompatibility of the materials used to immobilize enzymes on the surface of the electrode, which implicitly increases the stability of the enzyme and the biosensor during depositing. The modifications with nanoparticles of the electrode surface can reduce the over-potential of the compound oxidation and can increase the intensity of the oxidation current, which can considerably improve the sensitivity and selectivity of the determination [76].

Another challenge in developing biosensors is related to the interference of the matrix, which can affect their stability and sensitivity [136]. To resolve this problem, aspects related to the pre-treatment of the samples and to the optimization of the sensor sensitivity should be taken into consideration [137].

Ultimately, an important aspect of developing a biosensor based on enzymes is associated with selecting an adequate enzyme depending on the type of analyte to be analyzed. Due to the specificity of enzymatic reactions, one type of enzyme cannot detect all the antioxidants or evaluate the antioxidant properties of all the active compounds. For instance, laccase does not catalyze phenolic compounds with amino groups in meta position, such as the 3-amino phenol. Therefore, it would be of interest to develop various enzymatic biosensors which are adequate for detecting a particular type of antioxidant based on the action mechanisms of enzymes [138].

### 3.2. DNA-Based Biosensors

Biosensors based on DNA are considered an excellent alternative to determine the antioxidant activity of the compounds in various food samples, since these biosensors imitate the process and interactions which take place in the human body under oxidative stress. DNA can be immobilized on the surface of the transducer, using the genetic material as biological receiver. Nucleobases (adenine or guanine) are deteriorated by the presence of the operation system, resulting in the degradation of the electroanalytical signal. Adding a sample containing a polyphenol compound removes the ROS species, thus improving the response obtained. Consequently, this improvement can be correlated with the antioxidant activity of the respective sample [139]. During antioxidant monitoring through this type of biosensor, the DNA signal barely changes due to the ability of the antioxidant molecules to neutralize compounds that cause DNA damage. It is important to underline the fact that DNA damage is largely irreversible; thus, biosensors are generally single-use only. On the other hand, good reproducibility and constant sensitivity can be obtained [140].

The evaluation of antioxidants is mainly based on detecting DNA deterioration, since using electrochemical biosensors based on DNA for antioxidant evaluation is similar to the response of antioxidant activities in biological systems (usually simulating the damage caused by reactive oxygen species (ROS) in vivo) [141]. Single-stranded DNA (ssDNA) [142], double-stranded DNA (dsDNA) [143], as well as purine [144] and pyrimidine [145] bases, can serve as recognition elements for an electrochemical biosensor based on DNA. For DNA-based electrochemical sensors, any change in the oxidation peak of the DNA bases, before and after the interaction with the target molecule, will be evaluated. In the presence of antioxidant compounds, they compete with DNA for the hydroxyl radicals, which increase the DNA oxidation signal, indirectly determining the antioxidant activity of the analytes [146].

In this regard, the aim of a study was to develop a glassy carbon electrode by evaluating the total antioxidant activity of aromas and aromatic water, through immobilizing purine bases, adenine, and guanine. Square-wave voltammetry was used as the electrochemical method. The damages caused by the reactive oxygen species (ROS), namely the superoxide radical (O_2_^•−^) generated by the xanthine–xanthinoxidase (XOD) system on the surface of the biosensor, were evaluated. After adding active antioxidant compounds, it was found that the oxidative damages were reduced. Ascorbic acid, gallic acid, caffeic acid, p-coumaric acid, and resveratrol were used as antioxidants. These radicals are capable of reducing the superoxide radical, protecting the purine bases immobilized on the surface of the glassy carbon electrode. The results demonstrated that the biosensor based on DNA is adequate for the rapid evaluation of the total antioxidant activity in beverages [147].

Over three exhaustive papers, Labuda et al., described the use of a biosensor based on dsDNA to evaluate the total antioxidant activity of the polysaccharides in yeast [148], the phenolic compounds [149], and the flavanols and flavonols [150] in plant extracts. 

Another study involved the electrochemical determination of the interaction of quercetin with dsDNA, using two types of biosensors based on DNA to assess the damages caused by oxidized quercetin on DNA. Two different types of biosensors were prepared in order to study the interaction and observe modifications in the DNA film: a thick-layer dsDNA-modified GCE which requires a longer time of preparation and a thin-layer dsDNA-modified GCE obtained by the successive addition of dsDNA solution. The results showed that quercetin binds to dsDNA, and can be oxidized. The radicals formed during the oxidation of quercetin can break the hydrogen bonds in dsDNA, forming 8-oxoguanin. Thus, a mechanism is proposed for explaining the damages caused by oxidized quercetin on dsDNA immobilized on the surface of a glassy carbon electrode, also clarifying the formation of 8-oxoguanin. In conclusion, this study clearly demonstrated the importance of biosensors based on DNA in determining the mechanism of interaction between DNA and quercetin [151].

Sensitivity of the dsDNA structure towards OH’ radicals as the pro-oxidants has been utilized as the detection principle of an analytical procedure applied to the evaluation of antioxidant activity of six chlorogenic acids and extracts of ten coffees. A nanostructured electrochemical DNA-based biosensor was prepared using a commercial electrode assembly and treated in the DNA cleavage agent formed by the Fenton-type reaction [152].

Another method developed was to examine the DNA damage caused by dopamine and some ionic metals. Moreover, the inhibitory and restorative effects of some antioxidants, such as glutathione and ascorbic acid, were studied and compared using EIS and DPV. A pencil graphite electrode (PGE) was modified with MWCNTs and chitosan (CHIT), and then decorated with dsDNA (ds-DNA/CHIT–MWCNTs/PGE). Due to the interactions between dsDNA and the damaging agents (dopamine and metallic ions), the electrochemical and charge transfer properties of dsDNA on the surface of the electrode were modified, and these changes were observed through EIS and DPV. The study indicated that only dopamine, Cu(II), and Fe(III) cannot destroy DNA, and that dopamine + Cu(II) and dopamine + Fe(III) can deteriorate DNA. Furthermore, it was demonstrated that some antioxidants, such as glutathione and ascorbic acid, can exceed or reduce the influence of these damaging interactions to a minimum [153].

DNA-based biosensors using nanomaterials, with a large surface and improved electrochemical properties, have also been proposed. Thus, in a study carried out in 2016, guanine was selected as an electrochemical probe and was integrated with titanium oxide nanoparticles (TiO_2_) and with MWCNT on the surface of a GCE, in order to obtain an electrochemical biosensor (guanine/TiO_2_NP/MWCNTs/GCE) for the antioxidant evaluation of sodium pyrosulphate (Na_2_S_2_O_5_) (commonly used as antioxidant excipient for medicines and food preservatives) [145].

Silver is also used to immobilize dsDNA, due to its excellent conductivity, good electrocatalytic activity, and chemical stability [154]. When silver is modified on the surface of the electrode, very reactive silver oxides are generated, and they modify the conductivity of the immobilized dsDNA molecules. The conductor polymers incorporated with metallic particles offer an interesting system and represent a potential application for the sensors [155]. Wang et al., developed a sensor, using poly-L-glutamic acid, silver, and an exterior chitosan–dsDNA layer, to measure the total antioxidant activity of orange fruit beverages. Ascorbic acid and orange juice beverage could scavenge HO• and protect ds-DNA from damage effectively. Based on this, the antioxidant capacities of the orange juice beverage and ascorbic acid were studied by linear sweep voltammetry using Ru(NH_3_)_6_^3+^ (Hexaammineruthenium(III)) as indicator. The method had results which were comparable to those of the UV–vis method, indicating good stability and reproducibility [154].

Table 3 shows several examples of biosensors based on DNA for the determination of antioxidants in foods.

### 3.3. Advantages and Disadvantages of DNA-Based Biosensors

In addition to the benefits of DNA-based biosensors due to the nature of the DNA itself, there are a number of improvements that need to be considered when defining the performance of this type of biosensor. These are primarily related to DNA immobilization technology, which decreases the rate of electron transfer from the electrode surface. To select the immobilization technology, aspects related to the functional retention after DNA attachment, the chemical stability during all the post-testing stages, the orientation and the nature of biomolecular configuration, which need to be adequate, must be considered [156]. Moreover, due to the fact that a multitude of signals can be obtained in a matrix, the cross-contamination effects and the immobilization errors should be reduced to a minimum.

Another aspect is related to the specificity of these biosensors, as the complex characteristics of the sample matrix presents a great challenge going froward. The pentose in the nucleic acid has a significant specificity of the sequence and is considered a potential alternative for the oxidative detection of DNA [157].

Last but not least, the sensitivity of DNA-based biosensors is a very important characteristic which needs to be taken into account. Together with the development of nanotechnology and biotechnology, new nanostructured materials were used to increase the surface of the electrode, thus improving the sensitivity of these (bio)devices applied in quantifying the antioxidant activity. Furthermore, the miniaturization of the analytical procedure and the development of the lab-on-a-chip technique (which measures the various aspects related to the behavior of antioxidants on free radicals to generate a complete antioxidant profile in real time) can be useful [158].

## 4. Correlations between (Bio)sensors Responses and the Antioxidant Character of the Compounds

Antioxidant compounds can act as reducing agents in solutions, and have the tendency to be easily oxidated on the surface of the electrodes. Based on this fact, the relation between the chemical behavior of compounds with antioxidant properties and the resulting antioxidant activity is very interesting, since the low oxidation potential corresponds to a high antioxidant power [159]. On the other hand, the amperometric current and/or the charge measured in optimum oxidation conditions should provide information on extending their capacity, as well as on estimating their total potential. Furthermore, the oxidation potential helps to control selectivity so that most adequate conditions for measuring antioxidants and their antioxidant activity can be identified [160].

In general, correlation studies have been performed between electrochemical methods and commonly used antioxidant activity tests to highlight the possibility of using them as new tools in assessing the antioxidant activity. Since polyphenols are the main antioxidants involved in the antioxidant activity of various samples, studies on the correlation among electrochemical approaches were also carried out through the Folin-Ciocalteu method to attribute the antioxidant activity of polyphenols [124,127,131]. These studies demonstrate the antioxidant properties of foods and biological products due to the presence of polyphenol compounds and vitamins C and E. Along these lines, the concept of electrochemical index, defined as the total content of polyphenol compounds, obtained through the non-selective oxidation of all polyphenols, was introduced [60]. Through electrochemical methods, De Macêdo et al. [107] studied the antioxidant profile of the polyphenol compounds in red fruits, expressed as electrochemical index, and thereafter compared the results with those obtained through the DPPH spectrophotometric method, obtaining a good correlation between the content of polyphenol compounds and the antioxidant activity.

CV is one of the most common electrochemical methods in various studies performed to analyze redox systems. As described in the present paper, this method was applied to determine antioxidants in various food samples, but also in more complex samples, through sensors and biosensors. Moreover, these studies evaluated the correlation between the parameters obtained following electrochemical determinations (peak potential—E_p_, half-wave potential—E_1/2_, and I_p_—peak intensity), with results obtained through classical methods of evaluating antioxidant activity. Ricci et al. [84], on the one hand, and Photinon et al. [85], on the other hand, reported that the values of the peak currents in CV measurements are well correlated to the DPPH values. Furthermore, Firuzi et al. [86] noticed a good correlation between the peak currents and the antioxidant activity measured through the FRAP test in the case of flavonoids. Furthermore, the same correlation was described by G.K. Ziyatdinova et al. [87] in another study on individual spice antioxidant compounds (gallic acid, rosmarinic acid, capsaicin, thymol, and eugenol). Using a chemically modified electrode, Gualandi et al., determined the antioxidant activity of various compounds, usually considered antioxidants, and of various fruit juices, obtaining a good correlation between the data resulting from the electrochemical measurements and those resulting from the application of ORAC, DPPH, and ABTS methods [90].

SWV is another electrochemical method with excellent stability, adequate for analytical studies. This method was frequently used instead of chromatography to identify and to quantify antioxidants, such as quercetin, myricetin [88], and ascorbic acid [93]. Bordonaba et al. [88] demonstrated the possibility of using SWV and other electrochemical methods with screen-printed carbon electrodes to quantify and evaluate the antioxidant activity and the quantity of specific antioxidants, mainly polyphenols, in certain fruit juices. Later, the relation between the cumulative responses of the sensor to various potentials applied, and the total or individual antioxidants determined through conventional spectrophotometric methods (FRAP and Folin-Ciocalteu) were evaluated. Another study described the evaluation of the antioxidant activity of three flavonoids through the electrochemical method, using the glassy carbon electrode modified with graphene, doped with nitrogen, guanin, and polythionine, which helped to compare the results through the DPPH method. Good correlations were obtained for all the three studied compounds [93].

DPV is another electrochemical method which is adequate to characterize the redox behavior of antioxidants. Generally, the peak current is used to estimate the antioxidant activity or the concentration of antioxidants, while the peak potential can be used to identify the type of antioxidants [161]. Using this electrochemical method, Petković et al., determined the gallic acid in various wine samples, using an electrochemical sensor based on immobilizing the binuclear copper (II) octa azamacrocyclic complex, covered in graphite in a PVC matrix [89], while Souza et al., determined the same compound using a carbon paste electrode modified with carbon nanotubes [91]. The results obtained were compared to the Folin-Ciocalteu spectrophotometric method, achieving a calibration curve with standard gallic acid solutions. A good correlation between the two methods was obtained.

Taking into account the above, we can state that the electrochemical methods are among the most important approaches for evaluating the antioxidant activity, since they offer the possibility of measuring the electron transfer directly and rapidly, with good sensitivity and reproducibility [162]. These methods are direct, selective, and very sensitive, involving relatively low costs, and allow the analysis in various mediums, without the necessity of sample pre-treatment. Generally, the results obtained are very well correlated with the commonly used tests for determining antioxidants [163]. Generally, the performance of the electrochemical methods depends on the detection mechanism, the physical characteristics of the matrix analyzed (i.e., the nature of the electrolyte, the pH value, and the presence of interferent compounds), the sensor used, as well as the interaction between the antioxidant molecules and the functional groups of the electrode [164].

## 5. Conclusions

By taking into consideration the key role of antioxidants in treating diseases caused by oxidative stress, it is necessary to develop reliable tests in order to determine the antioxidant activity of various products, such as foods, supplements, or pharmaceutical drugs with a high antioxidant content.

In recent years, special attention was given to the determination of antioxidants, using sensors and/or biosensors due to the advantages of these methods, namely high sensitivity, ease of use and storage, rapid responses, easy automation, portability, and ease of miniaturization, which render them adequate for on-site diagnosis, thus reducing the risk of interference following the destabilization of compounds [165,166].

The combined efforts and achievements of screen printing and nanotechnology, biochemistry and electrochemistry, analytical chemistry, and organic polymers led to a new generation of sensors. The emergence and application of nanomaterials represent an integral part of sensors and their visible impact on research. The properties of nanomaterials are essential in developing a sensor, manifested in high electrochemical activity in comparison with that of the raw material [167]. This suggests that the transition from macro- systems to nano-scale systems significantly improves the characteristics of the sensors [168].

On the other hand, the importance of biosensors is growing constantly, since they help to integrate innovative materials and improve their performance, in terms of sensitivity and specificity [102,169]. At present, most research on improving the performances of biosensors is concentrated on developing new materials, especially conductor nanomaterials and functionalized polymers; however, the development and application of recombined biological compounds (enzymes and cells) are also of tremendous interest. The specific properties of nanoparticles (high capacity of adsorption, catalytic activity, excess of Gibbs free activation energy) render them very useful materials to be used in developing electrochemical sensors and biosensors [170].

In addition to this type of devices, the use of DNA-based biosensors is also preferred, since the measurement principle is closer to the activity of antioxidants in biological systems. ssDNA, dsDNA, or the nucleobases immobilized on the electrode are exposed to a radical attack similar to what takes place inside the cell, which can generate replication errors and a deceiving protein synthesis. The efficiency of neutralizing free radicals by antioxidants depends on the source of the free radicals. Taking this into account, in order to obtain a complete antioxidant profile, it is necessary to develop various analytical methodologies based on more free radical sources. For instance, it would be interesting to study the effect of reactive nitrogen or hydrogen peroxide on biosensors based on DNA. Therefore, these sensors are promising instruments for the rapid screening of the total antioxidant activity in various types of samples.

The large number of applications presented and discussed in the present paper clearly demonstrate the feasibility and utility of electrochemical sensors and biosensors for analyzing antioxidants in real samples, leading to complex matrices in their composition.

New directions in the development of biosensors by determining the antioxidant activity may be related to the use of multi-enzymatic systems, stabler and smaller immobilization platforms, the application of chemometric methods in the evaluation of experimental data, and the development of disposable biosensors.

## Figures and Tables

**Table 1:** Several examples of electrochemical assays based on sensors for the determination of antioxidants in food samples.

Nanomaterial (Sensor)	Antioxidants	Method	Linear Range (µM)	Limit of Detection (µM)	Real Sample	Ref.
GCE	Gallic acid Rosmarinic acid Capsaicin Thymol Eugenol	CV	19.8–1000 49.5–495 52.9–1060 60–200 3.74–1870	0.57–12 1.8–40	Spices	[87]
Graphite modified with [Cu_2_tpmc](ClO_4_)_4_ immobilized in PVC matrix	Gallic acid	DPV	2.5 × 10^−1^–100	1.48 × 10^−1^ 4.6	Wine samples	[89]
Carbon paste electrode modified with carbon nanotubes	Gallic acid	DPV	5.0 × 10^−1^– 15	3.0 × 10^−1^	Red and white wine	[91]
G/PTH/N-GPH/GCE	Myricetin Kaempferol Galangin Ascorbic acid	SWV	2.8–17	1.19	Fruit juices and plant extracts	[93]

SWV (square-wave voltammetry); [Cu_2_tpmc](ClO_4_)_4_ (dinuclear copper(II) octaazamacrocyclic N,N’,N’’,N’’’-tetrakis(2-pyridylmethyl)-1,4,8,11-tetraazacyclotetradecane complex); PVC (Poly(vinyl chloride)); AuNP (gold nanoparticles); G/PTH/N-GPH/GCE (electrochemical sensor based on guanine-, polythionine-, and nitrogen-doped graphene-modified glassy carbon electrode).

**Table 2:** Several examples of enzyme-based biosensors for the determination of antioxidants in food samples.

Receptor	Strategy	Detection Method	Target Molecule	Linear Range (µM)	LOD (µM)	Matrix	Ref.
Tyrosinase	Entrapment with water-soluble PVA, cross-linking using glutaraldehyde GA, cross-linking using GA and HSA	Amperometry	Catechol	0–109	26 ± 1	Infusions of: *Salvia microphylla* *Lippia dulcis* *Lippia alba*	[115]
	Tyrosinase immobilization onto a carbon paste electrode, in a Nafion film	Amperometry	p-hydroquinone	20–120	1.6	Red wine	[116]
Laccase	Laccase immobilization onto AuNPs/GNPI/SPCE	Amperometry	Hydroquinone	4–130	1.5	Blueberry syrup Wine	[123]
	Laccase immobilization onto AuNPs/Ppy/SPCE	Amperometry	Polyphenolic compounds	1–250	0.83	Propolis	[124]
	Tyrosinase or laccase immobilization on the surface of GCE modified with GO-MWCNTs hybrid	Amperometry	Catechol, gallic acid, pyrogallol, 1,2-dihydroxybenzoic acid, dopamine, epicatechin, rutin, caffeic acid, chlorogenic acid	1–340	Tyrosinase 0.5 Laccase 0.30	Fruit juice	[126]
Tyrosinase- Laccase	Bi-immobilization of laccase and tyrosinase phenoloxidase enzymes onto the electrode surface dopped with a mixture of the enzymes, glutaricdialdehyde and Nafion-ion exchanger	CA	Gallic acid Caffeic acid Ferulic acid (+)catechin (−)epicatechin	0.1–15.0 1.0 × 10^−2^ –2.0 3.0 × 10^−2^ –2.5 1.0 × 10^−2^–6.0 1.0 × 10^−2^ –9.0	19.0 × 10^−2^ 2.6 × 10^−2^ 6.4 × 10^−2^ 3.4 × 10^−2^ 4.3 × 10^−2^	Beer	[127]
	Modification of an ITO electrode with multiwalled carbon nanotubes, and co-entrapping the enzymes laccase and tyrosinase into a chitosan matrix	CA	Rosmarinic acid Caffeic acid Gallic acid	4.0 × 10^−^–6.4 4.0 × 10^−1^–7.4 1.6 × 10^−1^–8.1	2.50 × 10^−1^ 2.88 × 10^−1^ 1.55	extracts of *Salvia officinalis* cultures of *Basilicum callus*	[129]
Peroxidase	Immobilization of HRP and DNA onto silica–titanium	Amperometry	Chlorogenic acid	1–50	0.7	Coffee Tea	[131]

AuNPs/GNPI/SPCE (a gold nanoparticles–graphene nanoplatelet-modified screen-printed carbon electrode); AuNPs/Ppy/SPCE (gold nanoparticles electrodeposited in a screen-printed carbon electrode modified with polypyrrole) ITO (indium–tin oxide); GO-MWCNTs (graphene oxide and multi-walled carbon nanotubes); CA (chronoamperometry).

**Table 3:** Several examples of DNA-based biosensors for the determination of antioxidants in food samples.

Receptor	Strategy	Detection Method	Target Molecule	Linear Range (µM)	LOD (µM)	Matrix	Ref.
DNA	Immobilization of purine bases, guanine, and adenine on a GCE	SWV	Ascorbic acid Gallic acid Caffeic acid Coumaric acid Resveratrol	5.6–28.38 0.58–5.87 0.55–5.55 3.04–6.09 0.43–2.19	4.37 0.58 0.55 0.48 0.26	Beverages	[147]
	GCE modification with guanine/TiO_2_NPs/MWCNTs	DPV	Na_2_S_2_O_5_	1000–30,000	540	Adrenaline hydrochloride injection	[145]

## Data Availability

Not applicable.

## References

[1] Liu P., Li Y., Wang R., Ren F., Wang X. (2021). Oxidative Stress and Antioxidant Nanotherapeutic Approaches for Inflammatory Bowel Disease. Biomedicines.

[2] Demirci-Çekiç S., Özkan G., Avan A.N., Uzunboy S., Çapanoğlu E., Apak R. (2022). Biomarkers of Oxidative Stress and Antioxidant Defense. J. Pharm. Biomed. Anal..

[3] Sindhu R.K., Kaur P., Kaur P., Singh H., Batiha G.E.-S., Verma I. (2022). Exploring multifunctional antioxidants as potential agents for management of neurological disorders. Environ. Sci. Pollut. Res..

[4] Rahman M.M., Rahaman M.S., Islam M.R., Rahman F., Mithi F.M., Alqahtani T., Almikhlafi M.A., Alghamdi S.Q., Alruwaili A.S., Hossain M.S. (2022). Role of Phenolic Compounds in Human Disease: Current Knowledge and Future Prospects. Molecules.

[5] Karthikeyan A., Joseph A., Nair B.G. (2022). Promising bioactive compounds from the marine environment and their potential effects on various diseases. J. Genet. Eng. Biotechnol..

[6] Flieger J., Flieger W., Baj J., Maciejewski R. (2021). Antioxidants: Classification, Natural Sources, Activity/Capacity Measurements, and Usefulness for the Synthesis of Nanoparticles. Materials.

[7] Gupta E., Mishra P. (2021). Functional Food with Some Health Benefits, So Called Superfood: A Review. Curr. Nutr. Food Sci..

[8] Zulfiqar F., Ashraf M. (2022). Antioxidants as modulators of arsenic-induced oxidative stress tolerance in plants: An overview. J. Hazard. Mater..

[9] Rajput V.D., Singh R.K., Verma K.K., Sharma L., Quiroz-Figueroa F.R., Meena M., Gour V.S., Minkina T., Sushkova S., Mandzhieva S. (2021). Recent Developments in Enzymatic Antioxidant Defence Mechanism in Plants with Special Reference to Abiotic Stress. Biology.

[10] Filippov S.K., Domnina N., Vol’eva V. (2021). Future and the past of polymeric antioxidants. Polym. Adv. Technol..

[11] Rosa A.C., Bruni N., Meineri G., Corsi D., Cavi N., Gastaldi D., Dosio F. (2021). Strategies to expand the therapeutic potential of superoxide dismutase by exploiting delivery approaches. Int. J. Biol. Macromol..

[12] Munteanu I.G., Apetrei C. (2021). Analytical Methods Used in Determining Antioxidant Activity: A Review. Int. J. Mol. Sci..

[13] Mubinov A.R., Avdeeva E., Kurkin V.A., Latypova G.M., Farkhutdinov R.R., Kataev V.A., Ryazanova T.K. (2021). Fatty Acid Profile and Antioxidant Activity of Nigella Sativa Fatty Oil. Pharm. Chem. J..

[14] Spencer P.V., Libardi S.H., Dias F.F., Oliveira W.D.S., Thomasini R.L., Godoy H.T., Cardoso D.R., Junior S.B. (2021). Chemical Composition, Antioxidant and Antibacterial Activities of Essential Oil from *Cymbopogon densiflorus* (Steud.) Stapf Flowers. J. Essent. Oil Bear. Plants.

[15] Jongsawatsataporn N., Tanaka R. (2022). The Simultaneous Analysis of 14 Antioxidant Compounds Using HPLC with UV Detection and Their Application to Edible Plants from Asia. Food Anal. Meth..

[16] Wu Y., Gao H., Wang Y., Peng Z., Guo Z., Ma Y., Zhang R., Zhang M., Wu Q., Xiao J. (2022). Effects of different extraction methods on contents, profiles, and antioxidant abilities of free and bound phenolics of Sargassum polycystum from the South China Sea. J. Food Sci..

[17] Pizzo J.S., Cruz V.H.M., Rodrigues C.A., Manin L.P., Visentainer L., Santos O.O., Maldaner L., Visentainer J.V. (2022). Rapid determination of L-ascorbic acid content in vitamin C serums by ultra-high-performance liquid chromatography-tandem mass spectrometry. Int. J. Cosmetic Sci..

[18] Tartaglia A., Romasco T., D’Ovidio C., Rosato E., Ulusoy H., Furton K.G., Kabir A., Locatelli M. (2022). Determination of phenolic compounds in human saliva after oral administration of red wine by high performance liquid chromatography. J. Pharm. Biomed. Anal..

[19] Wei X., Chen J., Zhang X., Zhu Z., Liu H., Wang X., Guo X., Yang B. (2021). Organic Framework@Coordination Polymer Core-Shell Composites as Dual-Modal probe for Fluorescence and Colorimetric Analysis of Total Antioxidant Level in Saliva. Sens. Actuator B-Chem..

[20] Yuan L., Guo W., Fu Y., Zhang Z., Wang P., Wang J. (2021). A rapid colorimetric method for determining glutathione based on the reaction between cobalt oxyhydroxide nanosheets and 3,3’,5,5’-Tetramethylbenzidine. Microchem. J..

[21] Borah N., Tamuly C. (2022). Ultrasensitive Pd nano catalyst as peroxidase mimetics for colorimetric sensing and evaluation of antioxidants and total polyphenols in beverages and fruit juices. Talanta.

[22] Pilaquinga F., Morey J., Fernandez L., Espinoza-Montero P., Moncada-Basualto M., Pozo-Martinez J., Olea-Azar C., Bosch R., Meneses L., Debut A. (2021). Determination of Antioxidant Activity by Oxygen Radical Absorbance Capacity (ORAC-FL), Cellular Antioxidant Activity (CAA), Electrochemical and Microbiological Analyses of Silver Nanoparticles Using the Aqueous Leaf Extract of *Solanum mammosum* L.. Int. J. Nanomed..

[23] Bodó A., Radványi L., Kőszegi T., Csepregi R., Nagy D., Farkas Á., Kocsis M. (2021). Quality Evaluation of Light- and Dark-Colored Hungarian Honeys, Focusing on Botanical Origin, Antioxidant Capacity and Mineral Content. Molecules.

[24] Denardin C.C., Hirsch G.E., da Rocha R.F., Vizzotto M., Henriques A.T., Moreira J.C.F., Guma F.T.C.R., Emanuelli T. (2015). Antioxidant capacity and bioactive compounds of four Brazilian native fruits. J. Food Drug Anal..

[25] Poornima M.C., Salman M. (2021). Study of Antioxidant Properties and Phytochemical Constituents of *Sphagneticola trilobata* L. Leaves Extract. Int. J. Pharm. Sci. Res..

[26] Hofmann T., Albert L., Németh L., Vršanská M., Schlosserová N., Voběrková S., Visi-Rajczi E. (2021). Antioxidant and Antibacterial Properties of Norway Spruce (*Picea abies* H. Karst.) and Eastern Hemlock (*Tsuga canadensis* (L.) Carrière) Cone Extracts. Forests.

[27] Etienne O.K., Dall’Acqua S., Sinan K.I., Ferrarese I., Sut S., Sadeer N.B., Mahomoodally M.F., Ak G., Zengin G. (2021). Chemical characterization, antioxidant and enzyme inhibitory effects of Mitracarpus hirtus extracts. J. Pharm. Biomed. Anal..

[28] Pýnar S.M., Erez M.E., Fidan M., Eroðlu H., Dalar A. (2021). Determination of Biological Activity and Active Substances of Thecocarpus Carvifolius (BOISS.) Hedge & Lamond. Pharm. Chem. J..

[29] Borahan T., Girgin A., Atsever N., Zaman B.T., Chormey D.S., Bakırdere S. (2022). Development of a double-monitoring method for the determination of total antioxidant capacity as ascorbic acid equivalent using CUPRAC assay with RP-HPLC and digital image-based colorimetric detection. Eur. Food Res. Technol..

[30] Yalçın S., Karakaş Ö., Okudan E., Başkan K.S., Çekiç S.D., Apak R. (2021). HPLC Detection and Antioxidant Capacity Determination of Brown, Red and Green Algal Pigments in Seaweed Extracts. J. Chromatogr. Sci..

[31] Tel-Cayan G., Deveci E., Cayan F., Molo Z., Duru M.E., Yesil Y. (2022). Chemometrics Evaluation of Phytochemicals and Antioxidant Activities of the Extracts of Chaerophyllum Bulbosum Roots and Aerial Parts. Anal. Lett..

[32] Nickavar B., Malekitabar E. (2022). Compositional Analysis and Antioxidant Activities of Thymus pubescens Essential Oil from Iran. Comb. Chem. High Throughput Screen..

[33] Yang M., Yin M., Chu S., Zhao Y., Fang Q., Cheng M., Peng H., Huang L. (2022). Colour, chemical compounds, and antioxidant capacity of Astragali Radix based on untargeted metabolomics and targeted quantification. Phytochem. Anal..

[34] Tavares D.G., Guimarães S.D.S.C., Piccoli R.H., Duarte W.F., Cardoso P.G. (2022). *Arcopilus eremanthusum* sp. nov. as sources of antibacterial and antioxidant metabolites. Arch. Microbiol..

[35] Mahmoud O.A., Abdel_Hadi S.Y. (2022). Extraction and Purification of Lovastatin from the Edible Mushroom *Laetiporus sulphureus* and its Antioxidant Activity. Egypt. J. Bot..

[36] Abdulsattar J.O., Orabi M., Nasi Z.O. (2022). Phytochemical Profile, Antimicrobial, Antioxidant Activity and Cyclooxygenase 2 Inhibitory Properties of Nutmeg (Myristica Fragrans) Seeds Extract. Egypt. J. Chem..

[37] El Abdali Y., Agour A., Allali A., Bourhia M., El Moussaoui A., Eloutassi N., Salamatullah A.M., Alzahrani A., Ouahmane L., Aboul-Soud M.A.M. (2022). *Lavandula dentata* L.: Phytochemical Analysis, Antioxidant, Antifungal and Insecticidal Activities of Its Essential Oil. Plants.

[38] Felegyi-Tóth C.A., Garádi Z., Darcsi A., Csernák O., Boldizsár I., Béni S., Alberti Á. (2022). Isolation and quantification of diarylheptanoids from European hornbeam (*Carpinus betulus* L.) and HPLC-ESI-MS/MS characterization of its antioxidative phenolics. J. Pharm. Biomed. Anal..

[39] Martins G.R., Monteiro A.F., do Amaral F.R.L., da Silva A.S. (2021). A validated Folin-Ciocalteu method for total phenolics quantification of condensed tannin-rich acai (*Euterpe oleracea* Mart.) seeds extract. J. Food Sci. Technol. Mysore.

[40] Lidiková J., Čeryová N., Šnirc M., Vollmannová A., Musilová J., Tóthová M., Hegedȕsová A. (2021). Determination of bioactive components in selected varieties of pepper (*Capsicum* L.). Int. J. Food Prop..

[41] Luaces P., Pascual M., Pérez A.G., Sanz C. (2021). An Easy-to-Use Procedure for the Measurement of Total Phenolic Compounds in Olive Fruit. Antioxidants.

[42] Ieri F., Campo M., Cassiani C., Urciuoli S., Jurkhadze K., Romani A. (2021). Analysis of aroma and polyphenolic compounds in Saperavi red wine vinified in Qvevri. Food Sci. Nutr..

[43] Ilyasov I.R., Beloborodov V.L., Selivanova I.A., Terekhov R.P. (2020). ABTS/PP Decolorization Assay of Antioxidant Capacity Reaction Pathways. Int. J. Mol. Sci..

[44] Görüşük E.M., Bekdeşer B., Bener M., Apak R. (2020). ABTS radical-based single reagent assay for simultaneous determination of biologically important thiols and disulfides. Talanta.

[45] Phansi P., Tumma P., Thuankhunthod C., Danchana K., Cerdà V. (2021). Development of a Digital Microscope Spectrophotometric System for Determination of the Antioxidant Activity and Total Phenolic Content in Teas. Anal. Lett..

[46] Bibi Sadeer N., Montesano D., Albrizio S., Zengin G., Mahomoodally M.F. (2020). The Versatility of Antioxidant Assays in Food Science and Safety—Chemistry, Applications, Strengths, and Limitations. Antioxidants.

[47] Yalçın S., Uzun M., Karakaş Ö., Başkan K.S., Okudan E., Apak M.R. (2021). Determination of Total Antioxidant Capacities of Algal Pigments in Seaweed by the Combination of High-Performance Liquid Chromatography (HPLC) with A Cupric Reducing Antioxidant Capacity (CUPRAC) Assay. Anal. Lett..

[48] Gulcin İ. (2020). Antioxidants and antioxidant methods: An updated overview. Arch. Toxicol..

[49] Dall’Acqua S., Ak G., Sut S., Ferrarese I., Zengin G., Yıldıztugay E., Mahomoodally M.F., Sinan K.I., Lobine D. (2020). Phenolics from *Scorzonera tomentosa* L.: Exploring the potential use in industrial applications via an integrated approach. Ind. Crop. Prod..

[50] Kalinke C., Zanicoski-Moscardi A.P., de Oliveira P.R., Mangrich A.S., Marcolino-Junior L.H., Bergamini M.F. (2020). Simple and low-cost sensor based on activated biochar for the stripping voltammetric detection of caffeic acid. Microchem. J..

[51] David M., Florescu M., Bala C. (2020). Biosensors for Antioxidants Detection: Trends and Perspectives. Biosensors.

[52] Bounegru A.V., Apetrei C. (2020). Voltamperometric Sensors and Biosensors Based on Carbon Nanomaterials Used for Detecting Caffeic Acid—A Review. Int. J. Mol. Sci..

[53] Pwavodi P.C., Ozyurt V.H., Asir S., Ozsoz M. (2021). Electrochemical Sensor for Determination of Various Phenolic Compounds in Wine Samples Using Fe_3_O_4_ Nanoparticles Modified Carbon Paste Electrode. Micromachines.

[54] Sainz-Urruela C., Vera-López S., San Andrés M.P., Díez-Pascual A.M. (2021). Graphene-Based Sensors for the Detection of Bioactive Compounds: A Review. Int. J. Mol. Sci..

[55] Ivanova A., Gerasimova E., Gazizullina E. (2020). Study of Antioxidant Properties of Agents from the Perspective of Their Action Mechanisms. Molecules.

[56] Ziyatdinova G., Budnikov H. (2021). Analytical Capabilities of Coulometric Sensor Systems in the Antioxidants Analysis. Chemosensors.

[57] Petrucci R., Pasquali M., Scaramuzzo F.A., Curulli A. (2021). Recent Advances in Electrochemical Chitosan-Based Chemosensors and Biosensors: Applications in Food Safety. Chemosensors.

[58] Saikrithika S., Senthil Kumar A. (2020). Electrochemical Detections of Tea Polyphenols: A Review. Electroanalysis.

[59] Abeyrathne E.D.N.S., Nam K., Ahn D.U. (2021). Analytical Methods for Lipid Oxidation and Antioxidant Capacity in Food Systems. Antioxidants.

[60] Haque M.A., Morozova K., Ferrentino G., Scampicchio M. (2021). Electrochemical Methods to Evaluate the Antioxidant Activity and Capacity of Foods: A Review. Electroanalysis.

[61] Zhang J., Zhou Z., Kong Q. (2022). Progress in the Electrochemical Analysis of Flavonoids: A Scientometric Analysis in CiteSpace. Curr. Pharm. Anal..

[62] Della Pelle F., Compagnone D. (2018). Nanomaterial-Based Sensing and Biosensing of Phenolic Compounds and Related Antioxidant Capacity in Food. Sensors.

[63] Munteanu I.-G., Apetrei C. (2021). Electrochemical Determination of Chlorogenic Acid in Nutraceuticals Using Voltammetric Sensors Based on Screen-Printed Carbon Electrode Modified with Graphene and Gold Nanoparticles. Int. J. Mol. Sci..

[64] Nejad F.G., Tajik S., Beitollahi H., Sheikhshoaie I. (2021). Magnetic nanomaterials based electrochemical (bio)sensors for food analysis. Talanta.

[65] Ziyatdinova G., Guss E., Yakupova E. (2021). Electrochemical Sensors Based on the Electropolymerized Natural Phenolic Antioxidants and Their Analytical Application. Sensors.

[66] Chen R., Chen F., Sun M., Zhang R., Wu S., Meng C. (2021). Controllable synthesis and antioxidant activity of gold nanoparticles using chlorogenic acid. Inorg. Nano-Met. Chem..

[67] Ajaero C., Abdelrahim M.Y.M., Palacios-Santander J.M., Gil M.L.A., Naranjo-Rodríguez I., de Cisneros J.L.H.-H., Cubillana-Aguilera L.M. (2012). Comparative study of the electrocatalytic activity of different types of gold nanoparticles using Sonogel-Carbon material as supporting electrode. Sens. Actuators B Chem..

[68] Aghamirzaei M., Khiabani M.S., Hamishehkar H., Mokarram R.R., Amjadi M. (2021). Antioxidant, antimicrobial and cytotoxic activities of biosynthesized gold nanoparticles (AuNPs) from Chinese lettuce (CL) leave extract (*Brassica rapa var. pekinensis*). Mater. Today Commun..

[69] Ali S., Arthanari A., Shanmugam R. (2021). Antioxidant Activity of Silver Nanoparticles Synthesized Using Vetiveria zizanioides-In Vitro Study. J. Res. Med. Dent. Sci..

[70] Jayeoye T.J., Eze F.N., Olatunde O.O., Benjakul S., Rujiralai T. (2021). Synthesis of silver and silver@zero valent iron nanoparticles using *Chromolaena odorata* phenolic extract for antibacterial activity and hydrogen peroxide detection. J. Environ. Chem. Eng..

[71] Turunc E., Kahraman O., Binzet R. (2021). Green synthesis of silver nanoparticles using pollen extract: Characterization, assessment of their electrochemical and antioxidant activities. Anal. Biochem..

[72] García-Guzmán J.J., López-Iglesias D., Cubillana-Aguilera L., Bellido-Milla D., Palacios-Santander J.M., Marin M., Grigorescu S.D., Lete C., Lupu S. (2021). Silver nanostructures-poly(3,4-ethylenedioxythiophene) sensing material prepared by sinusoidal voltage procedure for detection of antioxidants. Electrochim. Acta.

[73] Singh S., Kumar U., Gittess D., Sakthivel T.S., Babu B., Seal S. (2021). Cerium oxide nanomaterial with dual antioxidative scavenging potential: Synthesis and characterization. J. Biomater. Appl..

[74] Abdelrahim M.Y.M., Benjamin S.R., Aguilera L.C., Naranjo-Rodriguez I., De Cisneros J.L.H.-H., Delgado J.J., Palacios-Santander J.M. (2013). Study of the Electrocatalytic Activity of Cerium Oxide and Gold-Studded Cerium Oxide Nanoparticles Using a Sonogel-Carbon Material as Supporting Electrode: Electroanalytical Study in Apple Juice for Babies. Sensors.

[75] Xie A., Wang H., Zhu J., Chang J., Gu L., Liu C., Yang Y., Ren Y., Luo S. (2021). A caffeic acid sensor based on CuZnO /MWCNTs composite modified electrode. Microchem. J..

[76] Brainina K., Stozhko N., Bukharinova M., Vikulova E. (2018). Nanomaterials: Electrochemical Properties and Application in Sensors. Phys. Sci. Rev..

[77] Bertel L., Miranda D.A., García-Martín J.M. (2021). Nanostructured Titanium Dioxide Surfaces for Electrochemical Biosensing. Sensors.

[78] Avelino K.Y., dos Santos G.S., Frías I.A., Silva-Junior A.G., Pereira M.C., Pitta M.G., de Araújo B.C., Errachid A., Oliveira M.D., Andrade C.A. (2021). Nanostructured sensor platform based on organic polymer conjugated to metallic nanoparticle for the impedimetric detection of SARS-CoV-2 at various stages of viral infection. J. Pharm. Biomed. Anal..

[79] Li J., Liu Z.-X., Li Y.-X., Shu G., Zhang X.-J., Marks R.S., Shan D. (2021). 2-Methylimidazole-assisted Morphology Modulation of a Copper-based Metal-organic Framework Transducer for Enhanced Electrochemical Peroxidase-like Activity. Electroanalysis.

[80] Beduk T., Filho J.I.D.O., Lahcen A.A., Mani V., Salama K.N. (2021). Inherent Surface Activation of Laser-Scribed Graphene Decorated with Au and Ag Nanoparticles: Simultaneous Electrochemical Behavior toward Uric Acid and Dopamine. Langmuir.

[81] Vinothkumar V., Koventhan C., Chen S.-M., Abinaya M., Kesavan G., Sengottuvelan N. (2021). Preparation of three dimensional flower-like cobalt phosphate as dual functional electrocatalyst for flavonoids sensing and supercapacitor applications. Ceram. Int..

[82] Lima A.P., dos Santos W.T.P., Nossol E., Richter E.M., Munoz R.A.A. (2020). Critical evaluation of voltammetric techniques for antioxidant capacity and activity: Presence of alumina on glassy-carbon electrodes alters the results. Electrochim. Acta.

[83] Abou Samra M., Chedea V.S., Economou A., Calokerinos A., Kefalas P. (2011). Antioxidant/prooxidant properties of model phenolic compounds: Part I. Studies on equimolar mixtures by chemiluminescence and cyclic voltammetry. Food Chem..

[84] Ricci A., Parpinello G.P., Teslić N., Kilmartin P.A., Versari A. (2019). Suitability of the Cyclic Voltammetry Measurements and DPPH• Spectrophotometric Assay to Determine the Antioxidant Capacity of Food-Grade Oenological Tannins. Molecules.

[85] Photinon K., Chalermchart Y., Khanongnuch C., Wang S.-H., Liu C.-C. (2010). A thick-film sensor as a novel device for determination of polyphenols and their antioxidant capacity in white wine. Sensors.

[86] Firuzi O., Lacanna A., Petrucci R., Marrosu G., Saso L. (2005). Evaluation of the antioxidant activity of flavonoids by “ferric reducing antioxidant power” assay and cyclic voltammetry. Biochim. Biophys Acta.

[87] Ziyatdinova G., Budnikov H. (2014). Evaluation of the antioxidant properties of spices by cyclic voltammetry. J. Anal. Chem..

[88] Bordonaba J.G., Terry L.A. (2012). Electrochemical behaviour of polyphenol rich fruit juices using disposable screen-printed carbon electrodes: Towards a rapid sensor for antioxidant capacity and individual antioxidants. Talanta.

[89] Petković B.B., Stanković D., Milčić M.K., Sovilj S.P., Manojlović D.D. (2015). Dinuclear copper(II) octaazamacrocyclic complex in a PVC coated GCE and graphite as a voltammetric sensor for determination of gallic acid and antioxidant capacity of wine samples. Talanta.

[90] Gualandi I., Ferraro L., Matteucci P., Tonelli D. (2015). Assessment of the Antioxidant Capacity of Standard Compounds and Fruit Juices by a Newly Developed Electrochemical Method: Comparative Study with Results from Other Analytical Methods. Electroanalysis.

[91] Souza L.P., Calegari F., Zarbin A.J.G., Marcolino-Júnior L.H., Bergamini M.F. (2011). Voltammetric determination of the antioxidant capacity in wine samples using a carbon nanotube modified electrode. J. Agric. Food Chem..

[92] David M., Şerban A., Popa C.V., Florescu M. (2019). A Nanoparticle-Based Label-Free Sensor for Screening the Relative Antioxidant Capacity of Hydrosoluble Plant Extracts. Sensors.

[93] Fu Y., You Z., Xiao A., Liu L., Zhou W. (2020). Electrochemical evaluation of the antioxidant capacity of natural compounds on glassy carbon electrode modified with guanine-, polythionine-, and nitrogen-doped graphene. Open Chem..

[94] Li C., Zhou Y., Ye B., Xu M. (2020). Sensitive Voltammetric Sensor for Evaluation of trans-resveratrol Levels in Wines based on Poly(L-lysine) Modified Electrode. J. Anal. Chem..

[95] Banica F., Bungau S., Tit D.M., Behl T., Otrisal P., Nechifor A.C., Gitea D., Pavel F.-M., Nemeth S. (2020). Determination of the Total Polyphenols Content and Antioxidant Activity of Echinacea Purpurea Extracts Using Newly Manufactured Glassy Carbon Electrodes Modified with Carbon Nanotubes. Processes.

[96] Koc T.B., Kuyumcu Savan E., Karabulut I. (2022). Determination of Antioxidant Properties and β-Carotene in Orange Fruits and Vegetables by an Oxidation Voltammetric Assay. Anal. Lett..

[97] Romero M.P.R., Brito R.E., Mellado J.M.R., González-Rodríguez J., Montoya M.R., Rodríguez-Amaro R. (2018). Exploring the relation between composition of extracts of healthy foods and their antioxidant capacities determined by electrochemical and spectrophotometrical methods. Lebensm.-Wiss. Technol..

[98] Pérez-López B., Merkoçi A. (2011). Nanomaterials based biosensors for food analysis applications. Trends Food Sci. Technol..

[99] Curulli A. (2020). Nanomaterials in Electrochemical Sensing Area: Applications and Challenges in Food Analysis. Molecules.

[100] Choleva T.G., Kappi F.A., Giokas D.L., Vlessidis A.G. (2015). Paper-based assay of antioxidant activity using analyte-mediated on-paper nucleation of gold nanoparticles as colorimetric probes. Anal. Chim. Acta.

[101] Ghalkhani M., Ghorbani-Bidkorbeh F. (2019). Development of Carbon Nanostructured Based Electrochemical Sensors for Pharmaceutical Analysis. Iran. J. Pharm. Res..

[102] Ye Y., Ji J., Sun Z., Shen P., Sun X. (2019). Recent advances in electrochemical biosensors for antioxidant analysis in foodstuff. TrAC Trends Anal. Chem..

[103] Wongkaew N., Simsek M., Griesche C., Baeumner A.J. (2019). Functional Nanomaterials and Nanostructures Enhancing Electrochemical Biosensors and Lab-on-a-Chip Performances: Recent Progress, Applications, and Future Perspective. Chem. Rev..

[104] Shao J., Wang C., Shen Y., Shi J., Ding D. (2022). Electrochemical Sensors and Biosensors for the Analysis of Tea Components: A Bibliometric Review. Front. Chem..

[105] Cesewski E., Johnson B.N. (2020). Electrochemical biosensors for pathogen detection. Biosens. Bioelectron..

[106] Serra B., Reviejo Á.J., Pingarrón J.M., Alegret S., Merkoçi A. (2007). Chapter 13 Application of electrochemical enzyme biosensors for food quality control. Comprehensive Analytical Chemistry.

[107] de Macêdo I.Y.L., Garcia L.F., Oliveira Neto J.R., de Siqueira Leite K.C., Ferreira V.S., Ghedini P.C., de Souza Gil E. (2017). Electroanalytical tools for antioxidant evaluation of red fruits dry extracts. Food Chem..

[108] Wee Y., Park S., Kwon Y.H., Ju Y., Yeon K.-M., Kim J. (2019). Tyrosinase-immobilized CNT based biosensor for highly-sensitive detection of phenolic compounds. Biosens. Bioelectron..

[109] Rodríguez-Delgado M.M., Alemán-Nava G.S., Rodríguez-Delgado J.M., Dieck-Assad G., Martínez-Chapa S.O., Barceló D., Parra R. (2015). Laccase-based biosensors for detection of phenolic compounds. TrAC Trends Anal. Chem..

[110] Stasyuk N., Gayda G., Zakalskiy A., Zakalska O., Serkiz R., Gonchar M. (2019). Amperometric biosensors based on oxidases and PtRu nanoparticles as artificial peroxidase. Food Chem..

[111] Cetó X., Céspedes F., Pividori M.I., Gutiérrez J.M., Del Valle M. (2012). Resolution of phenolic antioxidant mixtures employing a voltammetric bio-electronic tongue. Analyst.

[112] Zhang Z., Liu J., Fan J., Wang Z., Li L. (2018). Detection of catechol using an electrochemical biosensor based on engineered Escherichia coli cells that surface-display laccase. Anal. Chim. Acta.

[113] Agarwal P., Gupta R., Agarwal N. (2016). A Review on Enzymatic Treatment of Phenols in Wastewater. J. Biotechnol. Biomater..

[114] Nejadmansouri M., Majdinasab M., Nunes G., Marty J. (2021). An Overview of Optical and Electrochemical Sensors and Biosensors for Analysis of Antioxidants in Food during the Last 5 Years. Sensors.

[115] Rodríguez-Sevilla E., Ramírez-Silva M.-T., Romero-Romo M., Ibarra-Escutia P., Palomar-Pardavé M. (2014). Electrochemical Quantification of the Antioxidant Capacity of Medicinal Plants Using Biosensors. Sensors.

[116] Sýs M., Pekec B., Kalcher K., Vytřas K. (2013). Amperometric Enzyme Carbon Paste-Based Biosensor for Quantification of Hydroquinone and Polyphenolic Antioxidant Capacity. Int. J. Electrochem. Sci..

[117] Sýs M., Metelka R., Vytřas K. (2015). Comparison of tyrosinase biosensor based on carbon nanotubes with DPPH spectrophotometric assay in determination of TEAC in selected Moravian wines. Monatsh. Chem..

[118] Frangu A., Ashrafi A.M., Sýs M., Arbneshi T., Metelka R., Adam V., Vlček M., Richtera L. (2020). Determination of Trolox Equivalent Antioxidant Capacity in Berries Using Amperometric Tyrosinase Biosensor Based on Multi-Walled Carbon Nanotubes. Appl. Sci..

[119] Abosadeh D.J., Kashanian S., Nazari M., Parnianchi F. (2021). Fabrication of a Novel Phenolic Compound Biosensor Using Laccase Enzyme and Metal-organic Coordination Polymers. Anal. Bioanal. Chem. Res..

[120] Bounegru A.V., Apetrei C. (2021). Laccase and Tyrosinase Biosensors Used in the Determination of Hydroxycinnamic Acids. Int. J. Mol. Sci..

[121] García-Guzmán J.J., López-Iglesias D., Marin M., Lete C., Lupu S., Palacios-Santander J.M., Cubillana-Aguilera L., Inamuddin K.R., Mohammad A., Asiri A.M. (2019). Chapter 4—Electrochemical Biosensors for Antioxidants. Advanced Biosensors for Health Care Applications.

[122] de Oliveira Neto J.R., Rezende S.G., Lobón G.S., Garcia T.A., Macedo I.Y.L., Garcia L.F., Alves V.F., Torres I.M.S., Santiago M.F., Schmidt F. (2017). Electroanalysis and laccase-based biosensor on the determination of phenolic content and antioxidant power of honey samples. Food Chem..

[123] Zrinski I., Pungjunun K., Martinez S., Zavašnik J., Stanković D., Kalcher K., Mehmeti E. (2020). Evaluation of phenolic antioxidant capacity in beverages based on laccase immobilized on screen-printed carbon electrode modified with graphene nanoplatelets and gold nanoparticles. Microchem. J..

[124] Mohtar L.G., Aranda P., Messina G.A., Nazareno M.A., Pereira S.V., Raba J., Bertolino F.A. (2018). Amperometric biosensor based on laccase immobilized onto a nanostructured screen-printed electrode for determination of polyphenols in propolis. Microchem. J..

[125] García-Guzmán J.J., Hernández-Artiga M.P., de León L.P.-P., Bellido-Milla D. (2015). Selective methods for polyphenols and sulphur dioxide determination in wines. Food Chem..

[126] Vlamidis Y., Gualandi I., Tonelli D. (2017). Amperometric biosensors based on reduced GO and MWCNTs composite for polyphenols detection in fruit juices. J. Electroanal. Chem..

[127] ElKaoutit M., Naranjo-Rodriguez I., Temsamani K.R., de la Vega M.D., de Cisneros J.L.H.-H. (2007). Dual laccase—Tyrosinase based Sonogel—Carbon biosensor for monitoring polyphenols in beers. J. Agric. Food Chem..

[128] Montereali M., Della Seta L., Vastarella W., Pilloton R. (2010). A disposable Laccase–Tyrosinase based biosensor for amperometric detection of phenolic compounds in must and wine. J. Mol. Catal. B Enzym..

[129] Diaconu M., Litescu S.C., Radu G. (2011). Bienzymatic sensor based on the use of redox enzymes and chitosan–MWCNT nanocomposite. Evaluation of total phenolic content in plant extracts. Microchim. Acta.

[130] Steevensz A., Cordova Villegas L.G., Feng W., Taylor K.E., Bewtra J.K., Biswas N. (2014). Soybean peroxidase for industrial wastewater treatment: A mini review. J. Environ. Eng. Sci..

[131] Mello L.D., Sotomayor M.D.P.T., Kubota L.T. (2003). HRP-based amperometric biosensor for the polyphenols determination in vegetables extract. Sens. Actuators B Chem..

[132] Mello L.D., Alves A.A., Macedo D.V., Kubota L.T. (2005). Peroxidase-based biosensor as a tool for a fast evaluation of antioxidant capacity of tea. Food Chem..

[133] Mello L.D., Kubota L.T. (2014). Antioxidant capacity of Ilex paraguariensis extracts by using HRP-based biosensor. Lat. Am. Appl. Res..

[134] Munteanu I.G., Apetrei C. (2021). A Review on Electrochemical Sensors and Biosensors Used in Chlorogenic Acid Electroanalysis. Int. J. Mol. Sci..

[135] Tran T.T.T., Do M.N., Dang T.N.H., Tran Q.H., Le V.T., Dao A.Q., Vasseghian Y. (2022). A state-of-the-art review on graphene-based nanomaterials to determine antibiotics by electrochemical techniques. Environ. Res..

[136] Mei Y., He C., Zeng W., Luo Y., Liu C., Yang M., Kuang Y., Lin X., Huang Q. (2022). Electrochemical Biosensors for Foodborne Pathogens Detection Based on Carbon Nanomaterials: Recent Advances and Challenges. Food Bioprocess Technol..

[137] Song M., Lin X., Peng Z., Xu S., Jin L., Zheng X., Luo H. (2021). Materials and Methods of Biosensor Interfaces with Stability. Front. Mater..

[138] Roy J.J., Abraham T.E., Abhijith K.S., Kumar P.V.S., Thakur M.S. (2005). Biosensor for the determination of phenols based on Cross-Linked Enzyme Crystals (CLEC) of laccase. Biosens. Bioelectron..

[139] Thapa K., Liu W., Wang R. (2022). Nucleic acid-based electrochemical biosensor: Recent advances in probe immobilization and signal amplification strategies. Wiley Interdiscip. Rev.-Nanomed. Nanobiotechnol..

[140] Tandon A., Park S.H. (2021). DNA structures embedded with functionalized nanomaterials for biophysical applications. J. Korean Phys. Soc..

[141] Hashkavayi A.B., Hashemnia S., Osfouri S. (2020). Investigations of antioxidant potential and protective effect of Acanthophora algae on DNA damage: An electrochemical approach. Microchem. J..

[142] Tao S.-S., Wu G.-C., Zhang Q., Zhang T.-P., Leng R.-X., Pan H.-F., Ye D.-Q. (2019). TREX1 As a Potential Therapeutic Target for Autoimmune and Inflammatory Diseases. Curr. Pharm. Des..

[143] Ligaj M., Kobus-Cisowska J., Szczepaniak O., Szulc P., Kikut-Ligaj D., Mikołajczak-Ratajczak A., Bykowski P., Szymanowska D., Przeor M., Polewski K. (2021). Electrochemical screening of genoprotective and antioxidative effectiveness of *Origanum vulgare* L. and its functionality in the prevention of neurodegenerative disorders. Talanta.

[144] Barroso M.F., Delerue-Matos C., Oliveira M.B.P. (2012). Electrochemical evaluation of total antioxidant capacity of beverages using a purine-biosensor. Food Chem..

[145] Yue Y., Zhihong B., Sanming L., Kun Z. (2016). Electrochemical evaluation of antioxidant capacity in pharmaceutical antioxidant excipient of drugs on guanine-based modified electrode. J. Electroanal. Chem..

[146] Kamel A.H., Moreira F.T.C., Delerue-Matos C., Sales M.G.F. (2008). Electrochemical determination of antioxidant capacities in flavored waters by guanine and adenine biosensors. Biosens. Bioelectron..

[147] Barroso M.F., Delerue-Matos C., Oliveira M.B.P.P. (2011). Electrochemical DNA-sensor for evaluation of total antioxidant capacity of flavours and flavoured waters using superoxide radical damage. Biosens. Bioelectron..

[148] Bucková M., Labuda J., Sandula J., Krizková L., Stepánek I., Duracková Z. (2002). Detection of damage to DNA and antioxidative activity of yeast polysaccharides at the DNA-modified screen-printed electrode. Talanta.

[149] Labuda J., Bučková M., Heilerová L., Čaniová-Žiaková A., Brandšteterová E., Mattusch J., Wennrich R. (2002). Detection of Antioxidative Activity of Plant Extracts at the DNA-Modified Screen-Printed Electrode. Sensors.

[150] Labuda J., Bučková M., Heilerová Ľ., Šilhár S., Štepánek I. (2003). Evaluation of the redox properties and anti/pro-oxidant effects of selected flavonoids by means of a DNA-based electrochemical biosensor. Anal. Bioanal. Chem..

[151] Oliveira-Brett A.M., Diculescu V.C. (2004). Electrochemical study of quercetin—DNA interactions: Part II. In situ sensing with DNA biosensors. Bioelectrochemistry.

[152] Tomac I., Šeruga M., Labuda J. (2020). Evaluation of antioxidant activity of chlorogenic acids and coffee extracts by an electrochemical DNA-based biosensor. Food Chem..

[153] Ensafi A.A., Kazemnadi N., Amini M., Rezaei B. (2015). Impedimetric DNA-biosensor for the study of dopamine induces DNA damage and investigation of inhibitory and repair effects of some antioxidants. Bioelectrochemistry.

[154] Wang X., Jiao C., Yu Z. (2014). Electrochemical biosensor for assessment of the total antioxidant capacity of orange juice beverage based on the immobilizing DNA on a poly L-glutamic acid doped silver hybridized membrane. Sens. Actuators B Chem..

[155] Tsai T.-H., Lin K.-C., Chen S.-M. (2011). Electrochemical Synthesis of Poly(3,4-ethylenedioxythiophene) and Gold Nanocomposite and Its Application for Hypochlorite Sensor. Int. J. Electrochem. Sci. Int. J..

[156] Cagnin S., Caraballo M., Guiducci C., Martini P., Ross M., SantaAna M., Danley D., West T., Lanfranchi G. (2009). Overview of Electrochemical DNA Biosensors: New Approaches to Detect the Expression of Life. Sensors.

[157] Sassolas A., Leca-Bouvier B.D., Blum L.J. (2008). DNA Biosensors and Microarrays. Chem. Rev..

[158] Barroso M.F., de-los-Santos-Álvarez N., Delerue-Matos C., Oliveira M.B.P.P. (2011). Towards a reliable technology for antioxidant capacity and oxidative damage evaluation: Electrochemical (bio)sensors. Biosens. Bioelectron..

[159] Sochor J., Dobes J., Kryštofová O., Ruttkay-Nedecky B., Babula P., Pohanka M., Jurikova T., Zitka O., Adam V., Klejdus B. (2013). Electrochemistry as a Tool for Studying Antioxidant Properties. Int. J. Electrochem. Sci..

[160] Siddeeg A., AlKehayez N.M., Abu-Hiamed H.A., Al-Sanea E.A., AL-Farga A.M. (2021). Mode of action and determination of antioxidant activity in the dietary sources: An overview. Saudi J. Biol. Sci..

[161] Trofin A.E., Trincă L.C., Ungureanu E., Ariton A.M. (2019). CUPRAC Voltammetric Determination of Antioxidant Capacity in Tea Samples by Using Screen-Printed Microelectrodes. J. Anal. Methods Chem..

[162] Ávila M., Crevillén A.G., González M.C., Escarpa A., Hortigüela L.V., Carretero C.D.L., Martín R.A.P. (2006). Electroanalytical Approach to Evaluate Antioxidant Capacity in Honeys: Proposal of an Antioxidant Index. Electroanalysis.

[163] Olszowy-Tomczyk M. (2021). How to express the antioxidant properties of substances properly?. Chem. Pap..

[164] Pisoschi A.M., Cîmpeanu C., Predoi G. (2015). Electrochemical Methods for Total Antioxidant Capacity and its Main Contributors Determination: A review. Open Chem..

[165] Kumar A., Choudhary A., Kaur H., Mehta S., Husen A. (2021). Metal-based nanoparticles, sensors, and their multifaceted application in food packaging. J. Nanobiotechnol..

[166] Alhazmi H.A., Ahsan W., Mangla B., Javed S., Hassan M.Z., Asmari M., Al Bratty M., Najmi A. (2022). Graphene-based biosensors for disease theranostics: Development, applications, and recent advancements. Nanotechnol. Rev..

[167] Fatima A., Younas I., Ali M.W. (2022). An Overview on Recent Advances in Biosensor Technology and its Future Application. Arch. Pharm. Pract..

[168] Kalambate P.K., Noiphung J., Rodthongkum N., Larpant N., Thirabowonkitphithan P., Rojanarata T., Hasan M., Huang Y., Laiwattanapaisal W. (2021). Nanomaterials-based electrochemical sensors and biosensors for the detection of non-steroidal anti-inflammatory drugs. TrAC Trends Anal. Chem..

[169] Ribeiro D., Silva G.S., dos Santos D., Costa A.C., Ribeiro E.B., Badea M., Nunes G. (2021). Determination of the Antioxidant Activity of Samples of Tea and Commercial Sources of Vitamin C, Using an Enzymatic Biosensor. Antioxidants.

[170] Ziyatdinova G., Yakupova E., Davletshin R. (2021). Voltammetric Determination of Hesperidin on the Electrode Modified with SnO_2_ Nanoparticles and Surfactants. Electroanalysis.

